# Bipolar disorder and subsequent Parkinson's disease: a meta-analysis of cohort studies

**DOI:** 10.3389/fneur.2026.1825046

**Published:** 2026-06-05

**Authors:** Lijing Su, Xiaoling Feng, Yixia Chen, Zeqi Tang, Wen Shi, Yixian Qiu, Xiongtao Ma

**Affiliations:** 1 Department of Neurology, Ningbo Hospital of Integrated Traditional Chinese and Western Medicine, Ningbo, Zhejiang, China; 2 The Fifth School of Clinical Medical, Zhejiang Chinese Medical University, Hangzhou, Zhejiang, China; 3 Emergency Medical Center, Ningbo Hospital of Integrated Traditional Chinese and Western Medicine, Ningbo, Zhejiang, China

**Keywords:** bipolar disorder, eastern, incidence, meta-analysis, Parkinson's disease

## Abstract

**Background:**

The association between bipolar disorder (BD) and Parkinson's disease (PD) has been examined in several cohort studies, but existing evidence is limited by potential confounding and outcome misclassification. This meta-analysis aims to systematically evaluate this association while appraising the certainty of the evidence.

**Methods:**

A comprehensive literature search was conducted in PubMed, Web of Science, Embase and the Cochrane Library from database inception to April 2026. Cohort studies reporting PD risk in individuals with BD were eligible for inclusion. The outcome was the incidence of PD, with hazard ratios (HRs) and 95% confidence intervals (95% CIs) as effect measures. Random-effects models were employed and 95% prediction intervals (95% PIs) were calculated to reflect the expected range of true effects across studies. Effect estimates were extracted as reported, with varying degrees of adjustment across studies.

**Results:**

Six cohort studies fulfilled the predefined inclusion criteria. Pooled analysis demonstrated extreme heterogeneity (*I*^2^ = 92.7%, *P* < 0.001), with an HR of 3.65 (95% *CI* 2.16–6.17, *P* < 0.001, 95% *PI*, 0.67–20.00). Subgroup analyses were exploratory. Any observed differences by geographic region likely reflect variation in healthcare systems rather than biological effects. These findings are drawn from a limited number of observational studies with high risk of confounding and should be interpreted with caution.

**Conclusion:**

This meta-analysis suggests an association between BD and subsequent PD diagnosis, with very low certainty of evidence. The pooled estimate should not be interpreted as representing a single underlying effect, and a causal interpretation is not warranted due to uncontrolled medication exposure, outcome misclassification, and extreme heterogeneity.

## Introduction

Parkinson's disease (PD), the second most prevalent neurodegenerative disorder globally, affects over 6 million individuals worldwide, with its incidence and prevalence projected to rise dramatically-an estimated 25.2 million cases are expected by 2050 (1, 2). The mortality rate of PD has also increased substantially, from 1.76 per 100,000 people in 1994 to 5.67 per 100,000 in 2019 (3). Despite its significant public health burden, the etiopathogenesis of PD remains incompletely understood, and effective preventive strategies are lacking. Established risk factors for PD include genetic predisposition, environmental factors (lifestyle, diet, medication use) (4), type 2 diabetes (5), and mental disorders (6) such as bipolar disorder (BD).

BD is a chronic psychiatric condition characterized by recurrent episodes of mania/hypomania and depression, accompanied by profound alterations in cognition, behavior, and energy levels. The etiology of BD remains unclear, but genetic and environmental factors play pivotal roles in its pathogenesis (7). BD and PD share neurochemical and inflammatory pathophysiological pathways, including dopaminergic imbalance (8), immune dysregulation, and neurodegeneration (9, 10). Patients with BD display long-term disruption of dopaminergic homeostasis (11), which may gradually exhaust the compensatory capacity of dopaminergic neurons and progressively impair neuronal function. Furthermore, chronic low-grade inflammation in the brains of BD patients can lead to the loss of dopaminergic neurons (12, 13) and the pathological aggregation of α-synuclein (14), both of which result in neurodegeneration and eventually trigger the development of PD. A prior meta-analysis by Faustino et al. reported that BD increased the risk of subsequent PD (6). However, the meta-analysis by Faustino et al. contained three cross-sectional studies and lacked comprehensive subgroup analyses. Therefore, to further clarify the association between BD and PD, a systematic review and meta-analysis of cohort studies was conducted to evaluate the association between BD and subsequent PD incidence, informing clinical practice and early intervention.

## Methods

### Research registration

This research protocol had been registered on PROSPERO (CRD420251089392) and it was publicly accessible, conducted in conformity with Preferred Reporting Items for Systematic Reviews and Meta-Analyses (PRISMA) guidelines (15).

### Retrieval strategy

A systematic literature retrieval was carried out in several databases including PubMed, Web of Science, Embase and the Cochrane Library from the database inception to April 2026. Medical Subject Headings (MeSH) terms for “Parkinson Disease” and “Bipolar Disorder” were used as core search terms, with the full search strategy presented in the Supplementary Search Strategy. We included studies that investigated the risk of PD in patients with BD. To avoid missing relevant studies, we manually reviewed reference lists of eligible articles and relevant published systematic reviews. Literature screening was independently performed by two researchers to minimize the risk of selection bias. In case of disagreement regarding screening results, the third author was consulted to resolve the controversy.

### Inclusion and exclusion criteria

In accordance with the PICOS principle (16) (participants, interventions, comparisons, outcomes, and study design), the inclusion criteria were as follows: (a) participants: individuals without PD at the enrollment; (b) exposure: the diagnosis of BD; (c) comparison: no diagnosis of BD; (d) outcomes: the incidence of PD; (e) study design: cohort studies, including prospective and retrospective.

Studies with the following characteristics were excluded: (a) the articles not written in English; (b) not statistical data; (c) no interesting results; (d) the articles not published in full form (e.g., extraction, conference report); (e) updated research.

### Data extraction

The articles included in the meta-analysis had the data sorted and extracted according to the prepared table. The data included authors, year of publication, countries, the sources of data, the basic information of the participants (age and gender), the diagnostic bases of BD and PD, the numbers of subjects, follow-up times, the exclusion criteria and the adjusted factors. Data extraction was independently conducted by the same two researchers to minimize the risk of data extraction bias. In case of disagreement in the result of data extraction, the third author was consulted to resolve the controversy.

### Quality assessment

Risk of bias in the included non-randomized cohort studies was assessed primarily using the (ROBINS-I) (17), which is the current Cochrane-recommended instrument for this study design. The ROBINS-I evaluation covers seven domains: (1) bias due to confounding, (2) bias in selection of participants into the study, (3) bias in classification of interventions, (4) bias due to deviations from intended interventions, (5) bias due to missing data, (6) bias in measurement of outcomes, and (7) bias in selection of the reported result. Two researchers independently performed the ROBINS-I assessments. Any disagreements were resolved through discussion or, when necessary, by arbitration with a third author.

For transparency, the Newcastle-Ottawa Quality Assessment Scale (NOS) (18) was retained as a secondary quality assessment tool. The NOS checklist (18) evaluates three domains: selection, comparability, and outcomes. A maximum of nine points can be awarded (two points for comparability and one point for each remaining item). Studies scoring ≥7 points were classified as superior-quality research. The NOS was also applied independently by two researchers, with discrepancies resolved by the third author.

The certainty of evidence was assessed using the GRADE framework (19), considering risk of bias, inconsistency, indirectness, imprecision, and publication bias.

### Statistical analyses

Primary meta-analyses, subgroup interaction analyses, and forest plots were generated using the meta package (version 7.0-0) in R (version 4.5.3). Sensitivity analyses and funnel plots were conducted using STATA 12.0. The hazard ratios (HRs), 95% confidence intervals (95% CIs) and 95% prediction intervals (95% PIs) were employed to assess the association between BD and the incidence of PD. All results were analyzed using two-tailed tests, with *P* < 0.05 considered statistically significant. A lag analysis excluding PD events within 2–5 years after BD diagnosis was attempted. However, this was infeasible as none of the included studies applied a ≥2-year lag design.

Heterogeneity was assessed using the *I*^2^ statistic and Cochran's *Q*-test. An *I*^2^ < 25% corresponded to low heterogeneity, 25–50% to moderate heterogeneity, and >50% to high heterogeneity (20). Due to the inherent heterogeneity among the studies, random-effects models were adopted.

STATA 12.0 was employed for sensitivity analyses, including: (1) leave-one-out analysis for the main pooled estimate; (2) comparison of studies with vs. without adjustments for antipsychotic or mood stabilizer exposure to assess confounding by drug-induced parkinsonism (DIP); (3) leave-one-out analyses within subgroups.

Heterogeneity sources were explored by subgroup analyses. Pre-specified subgroups included geographic region and age at initial BD diagnosis. *Post-hoc* analyses included a 50-year age cutoff, NOS dichotomization (7 vs. 8–9), and follow-up duration (7-year cutoff: exploratory, supplementary). All subgroup analyses used random-effects models. Between-subgroup heterogeneity was assessed via Cochran's *Q*-test (meta package, version 7.0-0; R version 4.5.3), with *P* < 0.05 considered significant. Most subgroups contained only 2–3 studies, limiting credibility. Subgroup credibility was assessed using the Instrument for assessing the Credibility of Effect Modification Analyses (ICEMAN) (21) (Supplementary Table 4). For the geographic region subgroup, “Eastern” countries were defined as East Asian study sites (Taiwan, China and South Korea) and “Western” countries as Northern European and North American study sites (Denmark, the United Kingdom, and Canada). This classification was based on the locations of the included cohorts and was intended to reflect differences in healthcare systems, diagnostic coding practices, and antipsychotic prescribing patterns rather than biological or ethnic differences between populations.

With only six included studies, statistical power to detect publication bias was insufficient. A funnel plot is provided in the Supplementary Materials for descriptive purposes only (drawn by STATA 12.0).

## Results

### Study selection

**Figure 1:**
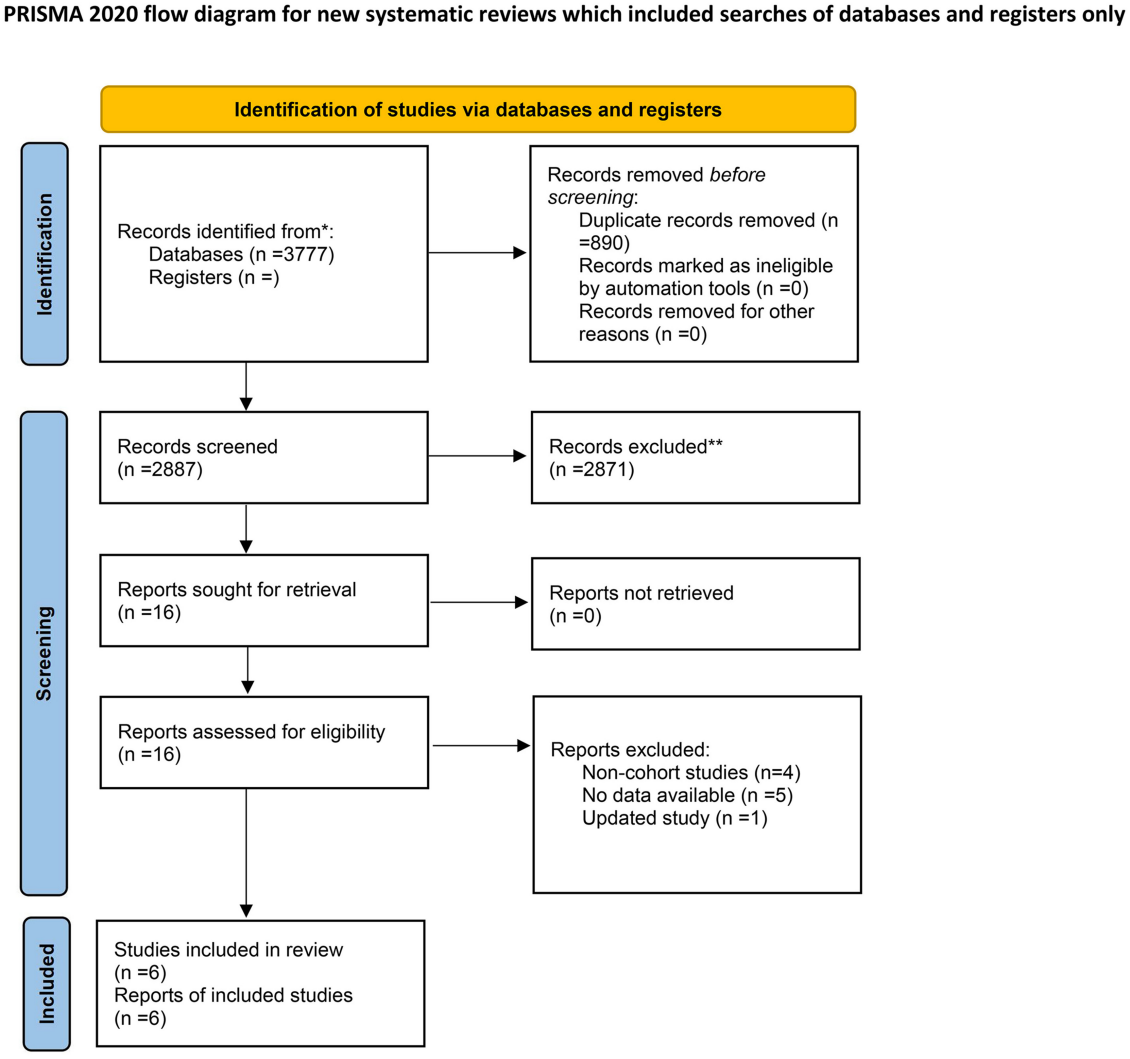
Flow diagram illustrating the study selection process. ^*^Consider, if feasible to do so, reporting the number of records identified from each database or register searched (rather than the total number across all database/registers). ^**^If automation tools were used, indicated how many were excluded by automation tools.

Based on the search strategy, 3,777 studies were obtained from the PubMed, Web of Science, Embase and Cochrane Library, of which 689 were excluded due to duplication, and 2,887 studies were reserved. After reading the title and abstract, 16 articles were kept up for full-text assessment. Finally, six studies met all inclusion criteria and were included in this analysis (22–27), 10 articles being excluded due to non-cohort studies (*n* = 4), no data available studies (*n* = 5) and an updated study (*n* = 1; Figure 1).

### Characteristics of the included study

Table 1 and Supplementary Table 1 showed characteristics of all the studies included in the meta-analysis containing four retrospective and two prospective studies. The publication years of the six articles ranged from 2001 to 2024. Geographically, these studies were distributed across different regions, with two conducted in Taiwan, China, one in Denmark, one in the UK, one in Korea and one in Canada, three from Eastern countries and three from Western countries. Regarding the sample sizes of the study groups, the number of diagnosed cases of BD was 44,714, while the number of participants in the control group was 9,979,654. For the diagnosis of BD, it was based on medical records, International Statistical Classification of Diseases and Related Health Problems-9-Clinical Modification (ICD-9-CM) codes, lithium medication use, International Statistical Classification of Diseases and Related Health Problems-10 (ICD-10) codes and symptoms assessed via the Structured Clinical Interview for DSM-IV Axis I Disorders. Meanwhile, the diagnosis of PD hinged primarily on record linkage diagnosis, ICD-9-CM codes, antiparkinsonian medication use, ICD-10 code (G20) and rare intractable diseases (V124). Table 1 also summarizes the handling of antipsychotic, mood stabilizer, and valproate exposure, washout periods, and PD diagnosis validation: most studies did not exclude or adjust for psychotropic drug exposure; PD ascertainment relied on administrative codes without specialist validation, and only one study applied a washout period. Additionally, there was variation in follow-up time across the studies, with durations spanning from 2 years to 17 years. Across these included studies, common adjustment factors contained age, gender, income, level of urbanization, smoking, alcohol, Charlson score, diabetes and hypertension. Of the six included studies, one (26) additionally reported stratified effect estimates by BD subtype. The adjusted HR for BD-I was 2.05 (95% *CI* 1.02-4.11) and for other BD was 3.33 (95%*CI* 2.22–4.11). As only a single study provided BD subtype–specific data, these estimates were not subjected to pooled meta-analysis.

**Table 1:** Characteristics of the studies included in the meta-analysis.

Author	Year	Country	Data sources	Antipsychotic exposure	Mood stabilizer exposure	Valproate exposure	Washout period (year)	The diagnosis of BD	The diagnosis of PD	PD diagnosis validation	Follow up (year)
Nilsson, F. M.	2001	Denmark	Denmark public hospital registration system	NA	NA	NA	No	Medical records	Record linkage diagnosis	Administrative codes only	17
Lin, H. L.	2014	Taiwan	Longitudinal health insurance database 2000 of Taiwan	NA	NA	NA	No	Record linkage diagnosis using ICD-9-CM codes 296.0, 296.4, 296.5, 296.6, 296.8	Medical records using ICD-9-CM code: 332.0	At least two concordant PD diagnoses	6
Mao-Hsuan Huang	2024	Taiwan	Taiwan National Health Insurance Research database	Analyzed in subgroup analysis	Excluded in sensitivity and analyzed in subgroup analysis	Analyzed in subgroup analysis	No	Record linkage diagnosis using ICD-9-CM codes: 296.0, 296.1, 296.4, 296.5, 296.6, 296.7, 296.80, 296.81, 296.89	Medical records using ICD-9-CM code: 332.0	Administrative codes confirmed by two physicians	2–10
Marras, C.	2016	Canada	Ontario administrative health care databases	Excluded in main analysis	Analyzed in subgroup analysis	Analyzed in subgroup analysis	No	Medical records via lithium medication	Record linkage diagnosis via anti parkinsonian medication	Administrative codes only	2
Xu, X.	2024	United Kingdom	UK biobank	Analyzed in subgroup analysis	Analyzed in subgroup analysis	NA	No	Record linkage diagnosis using ICD-10 and symptoms within the SCID-1	Medical records	Administrative codes only	13.8
Yoon, S. Y.	2024	Korea	Korean National Health Insurance Service and National Health Screening databases	NA	NA	NA	Yes (1)	Medical records	Medical records using ICD-10 code (G20) and registration code for RIDs (V124)	Administrative codes only	9

BD, bipolar disorder; PD, Parkinson's disease; ICD, international statistical classification of diseases and related health problems; CM, clinical modification; SCID, structured clinical interview for DSM IV axis I disorders; RIDs, rare intractable diseases; NA, not available; UK, United Kingdom.

### Quality assessment

Risk of bias in the six included cohort studies was assessed primarily using ROBINS-I. Three studies were rated as serious overall risk of bias and three as moderate overall risk of bias. Detailed assessments for each study are presented in Supplementary Table 2 and Supplementary Figure 1. For transparency, the NOS was retained as a secondary tool and all studies scored ≥7 points (Supplementary Table 3).

The results of GRADE showed very low certainty of evidence for the association between BD and PD due to serious risk of bias (uncontrolled antipsychotic exposure and outcome misclassification), extreme inconsistency, serious imprecision (Supplementary Table 5).

### Analysis of main outcome

**Figure 2:**
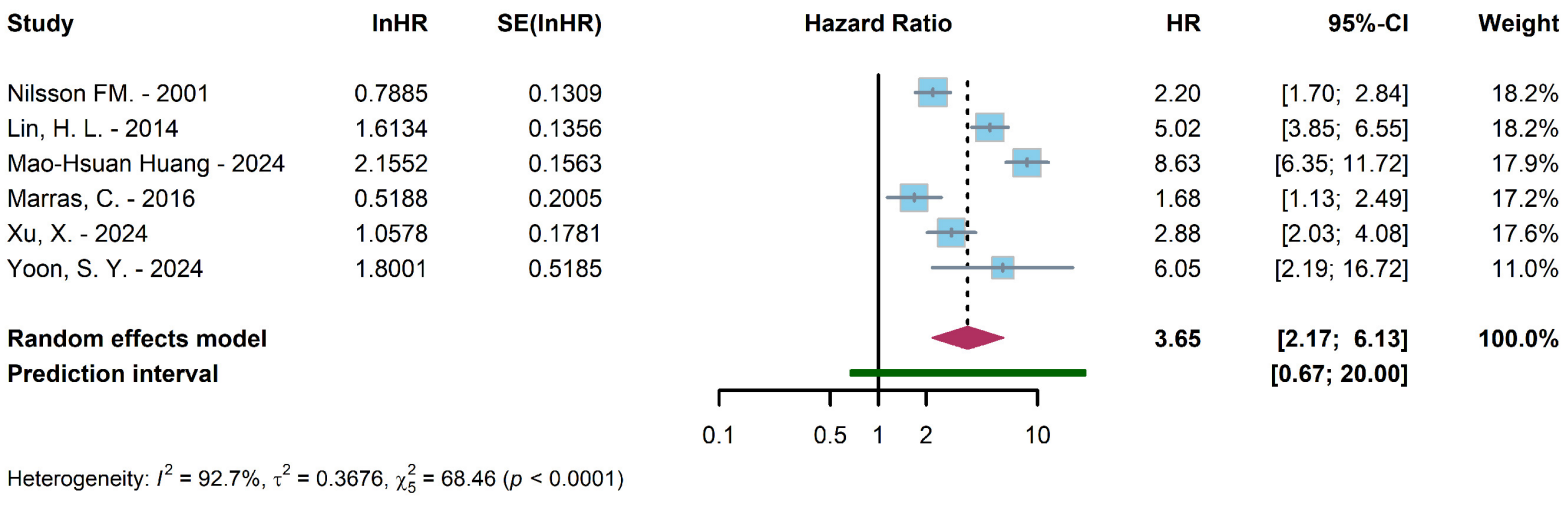
Forest plot of PD incidence in BD patients (*P* < 0.001).

Pooled analysis showed BD was associated with an increased incidence of subsequent PD [*HR* = 3.65, 95% *CI*, 2.16–6.17, *P* < 0.001, 95% *PI*, 0.67–20.00 (*I*^2^ = 92.7%, *P* < 0.001); Figure 2]. The wide PI and extreme heterogeneity indicate this estimate should not be interpreted as a single uniform underlying effect. The large magnitude likely reflects uncontrolled confounding by antipsychotic and mood stabilizer exposure, which can cause drug-induced parkinsonism indistinguishable from idiopathic PD in administrative databases.

A formal lag analysis excluding the first 2–5 years was infeasible due to lack of studies with ≥2-year lag design. Only Yoon 2024 applied a 1-year lag, reporting an HR of 6.05 (95% *CI* 2.19–16.72). Notably, this estimate was higher than the primary pooled estimate (HR 3.65), suggesting that a 1-year lag is insufficient to exclude drug-induced parkinsonism or that other methodological factors may contribute to the observed association. This is based on a single study and should be interpreted with caution.

### Sensitivity analyses and publication bias

**Figure 3:**
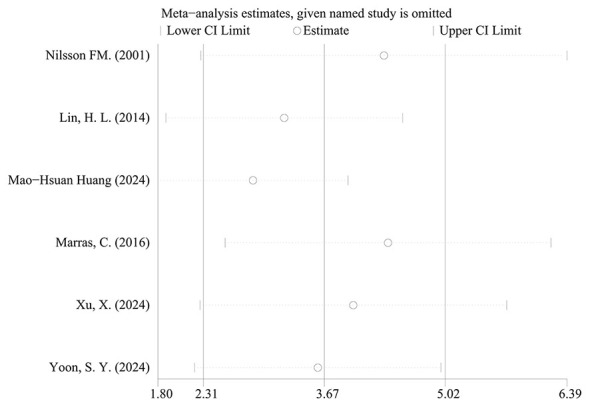
Sensitivity analysis to explore the risk of developing PD in patients with BD.

A sensitivity analysis was conducted to carry out detailed examination of each study one by one. Removing each individual study one by one, the results remained stable (Figure 3). Estimates were comparable between the two studies that adjusted for antipsychotic or mood stabilizer exposure and those with uncontrolled exposure. However, with only two studies in the adjusted group, this comparison is exploratory and cannot exclude drug-induced parkinsonism as a major confounder. Sensitivity analyses omitting one study at a time within subgroups yielded no substantial changes in direction; however, given that most subgroups comprised only 2–3 studies, these observations do not establish robust or credible subgroup effects.

Publication bias could not be reliably assessed due to the small number of included studies (*n* = 6), which is below the minimum of 10 studies recommended by Cochrane for meaningful funnel plot asymmetry assessment. The funnel plot is provided in the Supplementary Materials for descriptive purposes only (Supplementary Figure 2).

### Subgroup analyses

Given the high heterogeneity observed in the pooled analysis (*I*^2^ = 92.7%), subgroup analyses were conducted to explore potential sources of heterogeneity. Pre-specified subgroups included geographic region and age at initial BD diagnosis. *Post-hoc* analyses included gender, NOS score dichotomization, and follow-up duration (exploratory, supplementary only). All subgroup interaction tests were interpreted with caution: most subgroups contained only 2–3 studies, and formal ICEMAN assessment rated the credibility of all proposed effect modifications as very low credibility (Supplementary Table 4).

### Geographic region

**Figure 4:**
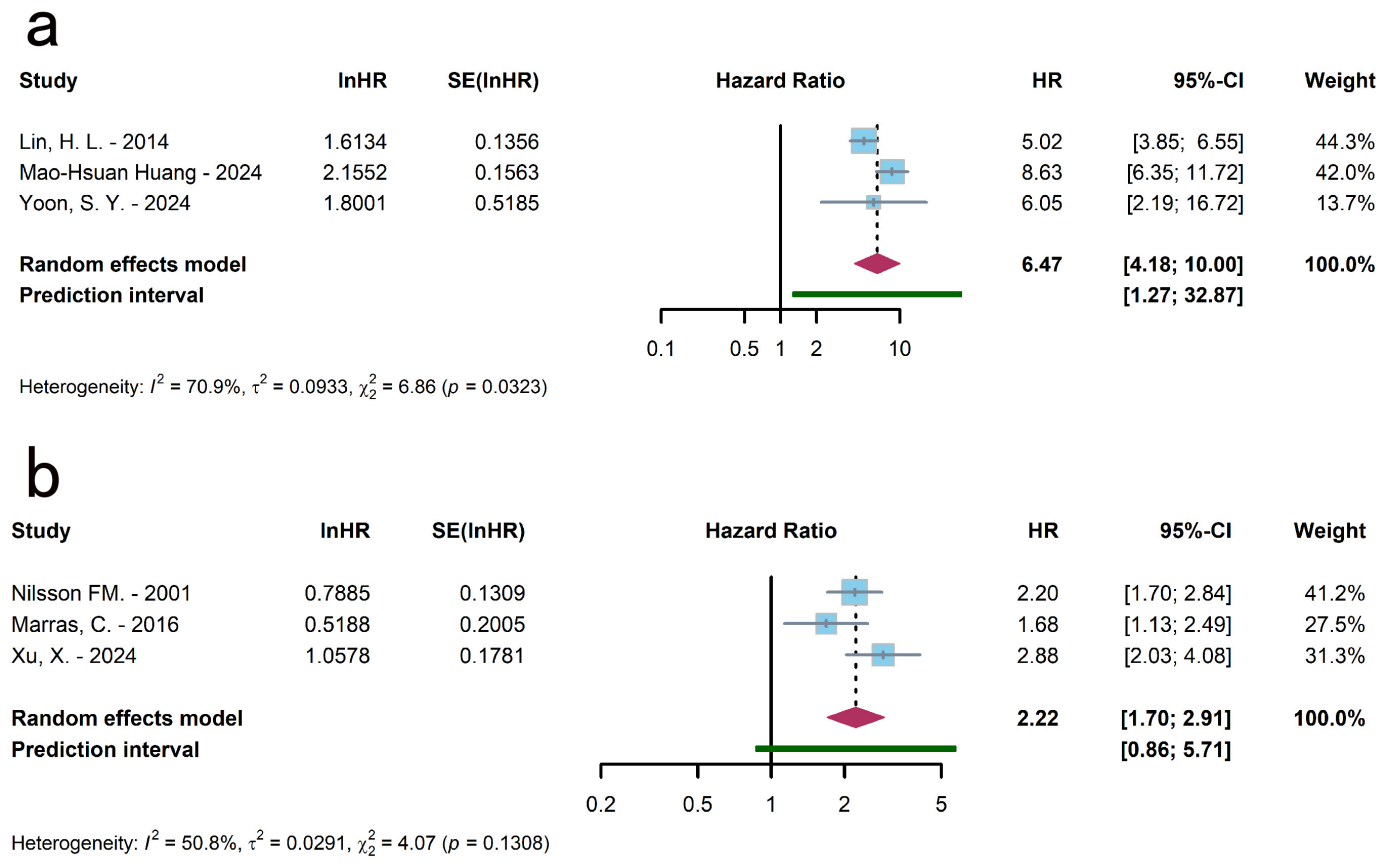
Forest plot of subgroup analysis of PD incidence in BD patients based on geographic region (**a**: Eastern countries: *P* < 0.001; **b**: Western countries: *P* < 0.001).

The incidence of PD was significantly higher in the BD group than in the control group in both Eastern and Western countries [Eastern: *HR* = 6.47, 95% *CI* 4.18–10.00, *P* < 0.001, 95% *PI* 1.27–32.87 (*I*^2^ = 70.9%, *P* = 0.0323); Western: *HR* = 2.22, 95% *CI* 1.70–2.91, *P* < 0.001, 95% *PI* 0.86–5.71 (*I*^2^ = 50.8%, *P* = 0.1308)]. A statistically significant subgroup interaction was observed (*P* < 0.001). However, because geographic region is completely confounded with healthcare system, diagnostic coding practice, and antipsychotic prescribing patterns, this interaction should not be interpreted as evidence of a true biological difference between populations (Figure 4).

### Gender

**Figure 5:**
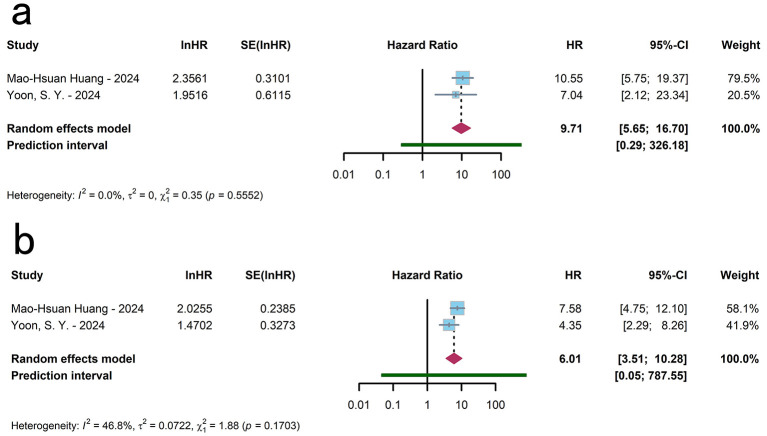
Forest plot of subgroup analysis of PD incidence in BD patients based on gender [**(a)**: female: *P* < 0.001; **(b)**: male: *P* < 0.001].

The incidence of PD was higher in the BD group compared to the control group in both sexes [males: *HR* = 9.71, 95%*CI* 5.65–16.70, *P* < 0.001, 95% *PI* 0.29–326.18 (*I*^2^ = 0%, *P* = 0.5552); females: *HR* = 6.01, 95% *CI* 3.51–10.28, *P* < 0.001, 95% *PI* 0.05–787.55 (*I*^2^ = 46.8%, *P* = 0.1703)], but no statistically significant difference was found between the two subgroups (*P* = 0.1320). The PIs for both sexes are extraordinarily wide and cross 1. This analysis was based on within-trial subgroup data from only two studies and was rated as having very low credibility by ICEMAN. The numerical difference in point estimates does not establish credible effect modification by gender (Figure 5).

### Age at initial BD diagnosis

**Figure 6:**
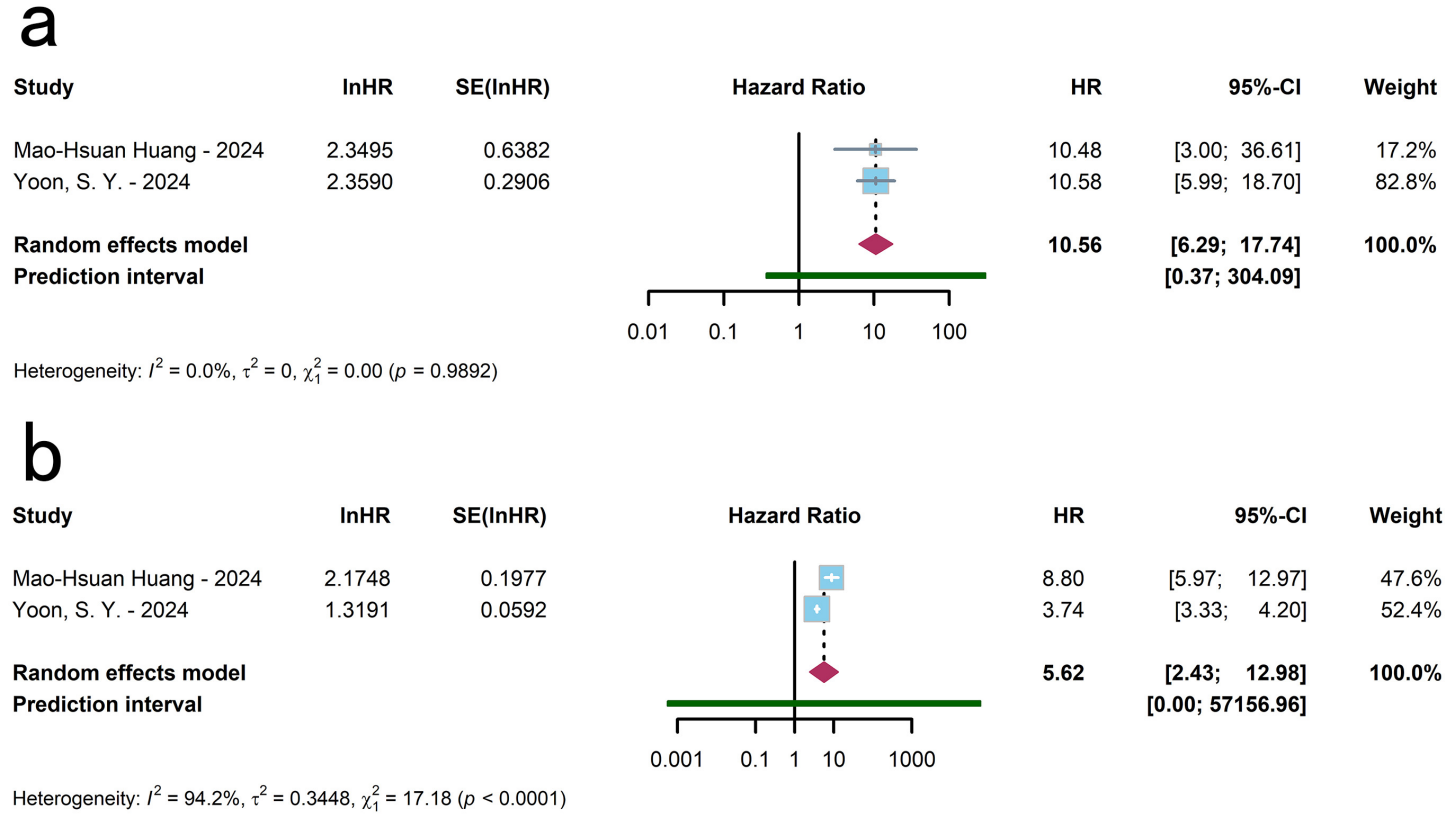
Forest plot of subgroup analysis of PD incidence in BD patients based on the initial diagnosis age of BD [**(a)**: < 50 years old: *P* < 0.001; **(b)**: >50 years old: *P* < 0.001].

A significant increased risk of PD was observed in both subgroups [age >50 years: *HR* = 5.62, 95% *CI* 2.43–12.98, *P* < 0.001, 95% *PI* 0.00–57,156.96 (*I*^2^ = 94.2%, *P* < 0.001); age < 50 years: *HR* = 10.56, 95%*CI* 6.29–17.74, *P* < 0.001, 95% *PI* 0.37–304.09 (*I*^2^ = 0%, *P* = 0.9892)], with no statistically significant difference between them (*P* = 0.140). Both PIs cross 1 and span several orders of magnitude, reflecting extreme uncertainty. This comparison was based on within-trial data from only two studies and was rated as very low credibility by ICEMAN. It cannot reliably assess whether age at BD onset modifies the association (Figure 6).

### Study quality (NOS score)

**Figure 7:**
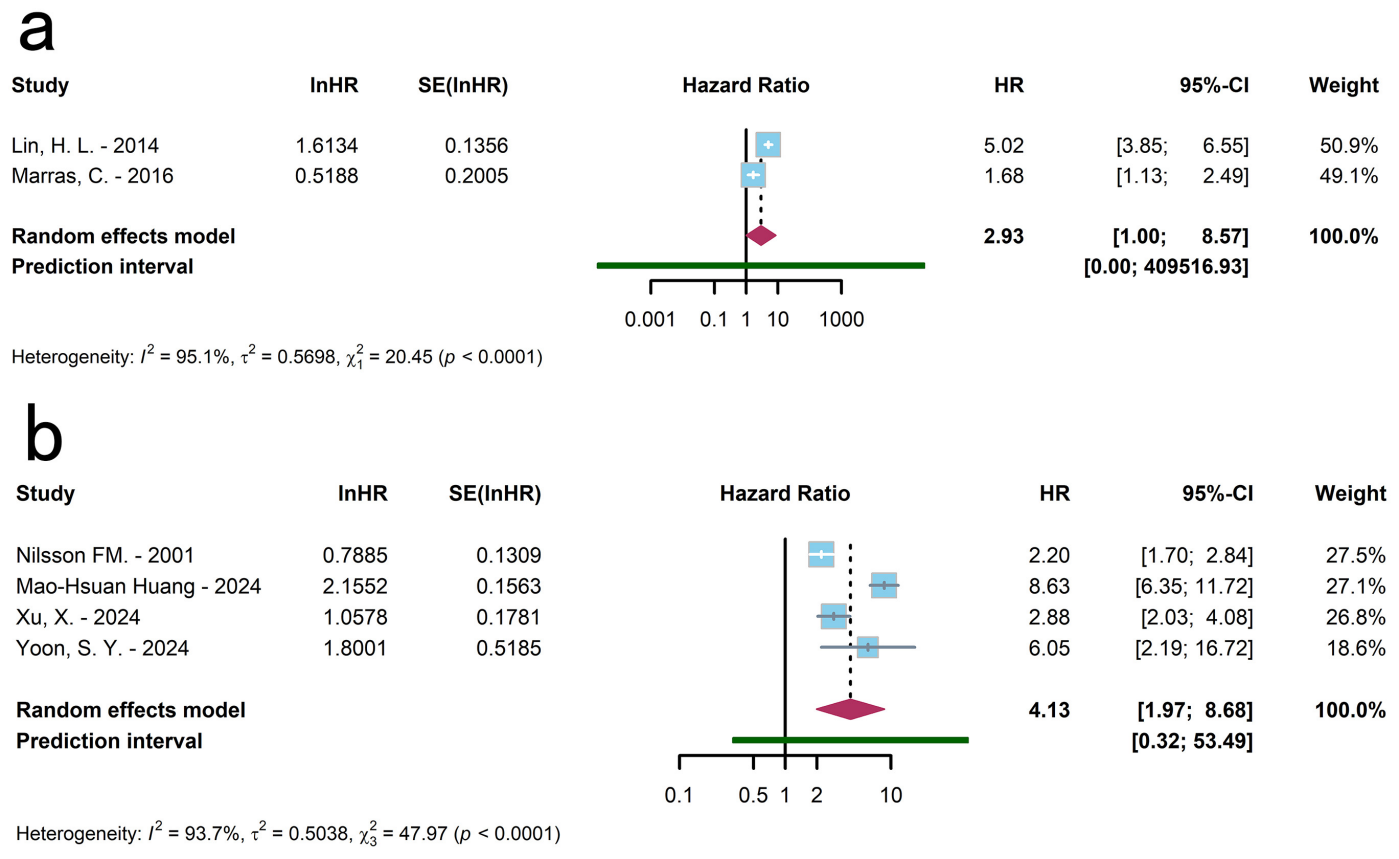
Forest plot of subgroup analysis of PD incidence in BD patients based on study quality [**(a)**: moderate-quality (*NOS* = 7): *P* = 0.049; **(b)**: high-quality (*NOS* = 8/9): *P* < 0.001].

Studies were dichotomized at *NOS* = 7 vs. 8–9 based on the median score across included studies. The incidence of PD was higher in the BD group than in the control group in both quality subgroups [*NOS* = 7: *HR* = 2.93, 95% *CI* 1.00–8.57, *P* = 0.049, 95% *PI* 0.00–409,516.93 (*I*^2^ = 95.1%, *P* < 0.001); *NOS* = 8/9: *HR* = 4.13, 95% *CI* 1.97–8.68, *P* < 0.001, 95% *PI* 0.32–53.49 (*I*^2^ = 93.7%, *P* < 0.001)], with no statistically significant difference between them (*P* = 0.597). The extremely wide PIs that cross 1 indicate that these quality-stratified estimates are not reliable summaries of a common underlying effect (Figure 7).

## Discussion

This meta-analysis identified a statistical association between BD and subsequent PD diagnosis in cohort studies. However, this estimate is affected by extreme heterogeneity and a high risk of confounding. The pooled HR should not be interpreted as an estimate of a single underlying effect. The wide PI reflects that the true association may vary substantially across healthcare settings and study designs. The discussion below first considers the most likely sources of bias and misclassification, before turning to-admittedly speculative-biological hypotheses.

The observed association may be largely explained by drug-induced parkinsonism (DIP) misclassified as idiopathic PD rather than by a true neurodegenerative causal pathway. Patients with BD are chronically exposed to antipsychotics, lithium, and valproate-all documented causes of DIP, which is clinically indistinguishable from idiopathic PD in administrative databases. Four of the six included studies did not adjust for these medications. The finding from the 2016 study by Marras et al. of increased anti-parkinsonian drug dispensing after lithium is most parsimoniously explained by lithium-associated tremor and diagnostic misclassification, not α-synucleinopathy. We therefore interpret the pooled estimate as reflecting measurement bias and confounding by indication rather than a causal effect. This interpretation does not exclude the existence of a true biological link, but testing such a hypothesis would require studies with validated diagnoses and controlled drug exposure—standards not met by the current evidence base.

### Mechanistic pathways (speculative)

Should a true causal link between BD and PD be established in future studies with validated diagnoses and controlled drug exposure, several biological hypotheses have been proposed. Central to these is dopaminergic system dysregulation: recurrent hyperdopaminergia during manic episodes and compensatory alterations during depressive phases could theoretically impair long-term neuronal resilience (11). Hypothesized molecular pathways include altered dopamine catabolism (e.g., catechol-O-methyltransferase variants) (28, 29) and dopamine receptor subtype 2 polymorphisms (30–34); immune dysfunction, chronic inflammation (12, 13, 35–41), and oxidative stress (42) have also been proposed. Inflammatory processes may further regulate α-synuclein metabolism (14). However, no immunological, oxidative, genetic, or biomarker data were analyzed in the present meta-analysis, and these mechanistic interpretations remain entirely hypothetical. A critical distinction must be made between two drug-related mechanisms: antipsychotics, lithium, and valproate can induce DIP, which is misclassified as idiopathic PD (a measurement bias); alternatively, these medications may theoretically contribute to genuine neurodegeneration through dopamine receptor blockade and mitochondrial impairment (42–45). The present data cannot distinguish between these pathways.

### Subgroup analyses

#### Geographic region

The subgroup analysis by geographic region showed higher observed effect estimates in studies from Eastern countries than in those from Western countries (subgroup interaction *P* < 0.001). However, this difference likely reflects variation in healthcare systems, diagnostic coding practices, and antipsychotic prescribing patterns rather than biological differences between populations (46, 47). The Eastern subgroup comprised administrative database studies from Taiwan and South Korea, while the Western subgroup included studies from Denmark, the UK, and Canada; these regions differ in case ascertainment methods, medication reimbursement policies, specialist referral pathways, and diagnostic cultures. We explicitly caution that this subgroup interaction test should not be interpreted as evidence of a true biological effect modifier.

#### Gender

The incidence of PD was higher in the BD group than in the control group in both sexes, but no statistically significant subgroup interaction was detected (*P* = 0.132). This analysis was based on within-trial subgroup data from only two studies and was rated as having very low credibility by ICEMAN; the numerical difference in point estimates does not establish credible effect modification by gender. Should a true difference exist, one speculative hypothesis is that estrogen may exert neuroprotective effects (48–50), although clinical trials of estrogen replacement in PD have yielded mixed or negative results, and the present analysis contains no hormonal data. Higher comorbidity burdens (51, 52) and potentially different medication dosing in male patients are alternative speculative explanations, but these were not directly measured in the included studies.

#### Initial diagnosis age of BD

A significantly increased risk of PD was observed in both age subgroups, with no statistically significant difference between groups (*P* = 0.140). This comparison was rated as having very low credibility by ICEMAN, owing to the inclusion of only two studies; therefore, it cannot reliably assess whether age at BD onset modifies the association. One speculative hypothesis is that younger BD patients may require longer durations of pharmacotherapy, resulting in greater cumulative antipsychotic exposure (53). However, this hypothesis was not examined in the present analysis.

#### Study quality (NOS score)

Studies were dichotomized by NOS score (7 vs. 8–9) based on the median value across included studies. Although the direction of association remained consistent across quality subgroups, the lack of significant subgroup interaction (*P* = 0.597), wide CIs, small study count, and *post-hoc* cutoff definition indicate that this consistency cannot confirm the robustness of the main findings.

#### Follow-up duration (exploratory)

This analysis was exploratory and is presented in the Supplementary Materials (Supplementary Figure 3). No significant subgroup interaction was identified (*P* = 0.889). These findings should be interpreted with caution, owing to the arbitrary *post-hoc* cutoff and very low credibility per the ICEMAN framework.

#### Consideration of the unusually high effect size

The pooled HR of 3.65 and subgroup HRs of up to 9.71 in males, 10.56 in early-onset BD patients, and 6.47 in Eastern populations are markedly larger than effect sizes for any established non-genetic risk factor for PD. For context, type 2 diabetes is associated with an HR of approximately 1.2–1.4 (54), and pesticide exposure confers an odds ratio of approximately 1.5–2.0 (55). An HR near 10 is comparable to or even greater than the effect size associated with major pathogenic mutations such as Leucine-Rich Repeat Kinase 2, Glycine 2019 Serine (56). In observational research, effect sizes of this magnitude most commonly arise from uncontrolled confounding, selection bias, and outcome misclassification rather than true causal effects. Accordingly, our findings require extremely cautious interpretation, and we have deliberately tempered causal inferences throughout the manuscript. These strong associations should be regarded as epidemiological links rather than definitive proof of causality.

#### Comparison with previous studies

Consistent with previous research, our findings align with a prior meta-analysis by Faustino et al., who first reported an elevated PD risk among patients with BD. However, several notable differences exist between the two studies. Importantly, the earlier meta-analysis incorporated three cross-sectional studies, which restrict causal inference and prevent clear clarification of the temporal relationship between BD and PD. Furthermore, that study lacked comprehensive subgroup analyses to explore potential effect modifiers. In contrast, the present meta-analysis exclusively included cohort studies with adjusted HRs and performed subgroup analyses, alongside formal interaction tests and ICEMAN-based credibility evaluation. Although these methodological improvements enhance analytical transparency, key limitations of the current evidence base remain unresolved, including extreme heterogeneity (95% *PI*: 0.67–20.00), substantial confounding related to psychotropic medication exposure, and a limited number of eligible studies. Overall, the present results support a temporal association between BD and subsequent PD onset, but cannot confirm a causal relationship.

#### Limitations

Our study has several limitations. First, the number of included studies was relatively limited (*n* = 6), which restricted statistical power, precluded meta-regression, and rendered most subgroup analyses unreliable, as the majority were based on only 2–3 studies. Second, considerable heterogeneity was identified across included studies (*I*^2^ = 92.7%), and the 95% *PI* (0.67–20.00) indicated that the true effect may range from protective to strongly harmful across different settings. Third, stratified data on BD subtypes (BD-I vs. other BD) were available from only one included study (27), precluding pooled subtype-specific risk analyses. BD-I and BD-II differ in pharmacotherapy regimens and may carry distinct risks of drug-induced parkinsonism. Detailed data on PD subtypes (idiopathic vs. drug-induced) were unavailable across all studies, preventing further subtype-specific risk analyses. Fourth, the 95%PIs for the sex, age-at-onset of BD, and NOS = 7 subgroups were extremely wide and crossed the null value of 1 (ranging from 0.05 to 787.55, 0.00 to 57,156.96, and 0.00 to 409,516.93, respectively), indicating severe imprecision because each subgroup included only two studies. Accordingly, these estimates are not robust enough to support credible effect modification and should be interpreted with great caution to avoid overinterpretation. Fifth, a formal lag analysis excluding the initial 2–5 years following BD diagnosis was infeasible, since no included study adopted a lag period of ≥2 years by design. The single study with a 1-year lag (27) reported an HR of 6.05 (95% *CI*: 2.19–16.72). This point estimate was elevated, rather than attenuated, relative to the primary pooled result, suggesting that a 1-year lag is inadequate to eliminate persistent drug effects or that unmeasured confounding operates throughout the entire follow-up duration. Reverse causation represents an additional key concern: prodromal PD may manifest with psychiatric symptoms that are misdiagnosed as BD, especially among older adults, and the absence of an adequate lag period cannot rule out this scenario. Accordingly, the observed BD-PD association may be bidirectional and requires clarification through future studies with validated diagnostic criteria and extended washout periods. Finally, protopathic bias, surveillance bias, and residual confounding by unmeasured psychotropic medication exposure remain important unresolved alternative explanations. Collectively, these shortcomings may weaken the robustness of our pooled estimates, which necessitates highly cautious interpretation. Further large-scale, methodologically rigorous, multi-ethnic studies are warranted to verify these findings.

## Conclusion

In summary, this meta-analysis of six cohort studies identified an association between BD and subsequent PD diagnosis. However, the overall certainty of this evidence is very low. The wide PI suggests substantial variability in the true effect across different clinical and healthcare settings, and the pooled HR should not be regarded as a uniform underlying effect size. The observed association is largely driven by uncontrolled confounding related to antipsychotic and mood stabilizer use, as well as frequent misclassification of drug-induced parkinsonism as idiopathic PD within administrative healthcare databases. Although subgroup analyses showed consistent directional trends, each comparison relied on merely 2–3 studies and was graded as very low credibility per the ICEMAN framework. Geographic disparities in effect estimates most likely stem from differences in healthcare delivery and prescribing practices, rather than inherent biological disparities across populations. Given prominent heterogeneity and methodological limitations, the true magnitude of the BD-PD association remains unclear, and causal inference is not justified based on the current evidence base. Future well-designed prospective cohort studies with validated diagnostic ascertainment, comprehensive adjustment for psychotropic medication exposure, and structured lag analyses are essential before deriving any clinical or public health recommendations. Proposed biological mechanisms underlying this relationship remain speculative. Large-scale, multi-ethnic collaborative research is also required to enhance the generalizability of relevant findings.

## Supplementary material

Supplementary Figure 1 ROBINS-I traffic-light plot of risk of bias across seven domains for the six included studies. **Red**, serious; **yellow**, moderate; **green**, low.

Supplementary Figure 2 Funnel plot of the six included studies. With fewer than 10 studies, statistical power to detect asymmetry is insufficient; this plot is provided for descriptive purposes only and should not be interpreted as evidence of publication bias.

Supplementary Figure 3 Forest plot of subgroup analysis of PD incidence in BD patients based on follow-up time (**a**: < 7 years: *P* = 0.049; **b**: >7 years: *P* < 0.001).

Supplementary Table 1 Characteristics of the studies included in the meta-analysis.

Supplementary Table 2 Risk of bias assessment of ROBINS-I (risk of bias judgements in non-randomized studies of interventions).

Supplementary Table 3 Quality assessment of cohort studies included.

Supplementary Table 4 ICEMAN assessment of the credibility of subgroup analyses.

Supplementary Table 5 GRADE summary of findings: certainty of evidence for the association between bipolar disorder and subsequent Parkinson's disease diagnosis.

Supplementary Search Strategy Detailed search strategy for PubMed, web of science, Embase and Cochrane library.

## References

[1] Tolosa E Garrido A Scholz SW Poewe W. Challenges in the diagnosis of Parkinson's disease. Lancet Neurol. (2021) 20:385–97. doi: 10.1016/S1474-4422(21)00030-2 33894193 PMC8185633

[2] Su D Cui Y He C Yin P Bai R Zhu J . Projections for prevalence of Parkinson's disease and its driving factors in 195 countries and territories to 2050: modelling study of Global Burden of Disease Study 2021. BMJ. (2025) 388:e080952. doi: 10.1136/bmj-2024-080952 40044233 PMC11881235

[3] Lampropoulos IC Malli F Sinani O Gourgoulianis KI Xiromerisiou G. Worldwide trends in mortality related to Parkinson's disease in the period of 1994–2019: analysis of vital registration data from the WHO mortality database. Front Neurol. (2022) 13:956440. doi: 10.3389/fneur.2022.956440 36267881 PMC9576872

[4] Bellou V Belbasis L Tzoulaki I Evangelou E Ioannidis JP. Environmental risk factors and Parkinson's disease: an umbrella review of meta-analyses. Parkinsonism Relat Disord. (2016) 23:1–9. doi: 10.1016/j.parkreldis.2015.12.008 26739246

[5] Han K Kim B Lee SH Kim MK. A nationwide cohort study on diabetes severity and risk of Parkinson disease. NPJ Parkinsons Dis. (2023) 9:11. doi: 10.1038/s41531-023-00462-8 36707543 PMC9883517

[6] Faustino PR Duarte GS Chendo I Castro Caldas A Reimão S Fernandes RM . Risk of developing Parkinson disease in bipolar disorder: a systematic review and meta-analysis. JAMA Neurol. (2020) 77:192–8. doi: 10.1001/jamaneurol.2019.3446 31609378 PMC6802493

[7] Vieta E Berk M Schulze TG Carvalho AF Suppes T Calabrese JR . Bipolar disorders. Nat Rev Dis Primers. (2018) 4:18008. doi: 10.1038/nrdp.2018.8 29516993

[8] Geelhand de Merxem R Launay S Hanak C. Association between bipolar disorder and Parkinson's disease. Psychiatr Danub. (2023) 35:66–71. doi: 10.1007/s100720200073 37800205

[9] Jones GH Vecera CM Pinjari OF Machado-Vieira R. Inflammatory signaling mechanisms in bipolar disorder. J Biomed Sci. (2021) 28:45. doi: 10.1186/s12929-021-00742-6 34112182 PMC8194019

[10] Karabulut S Taşdemir I Akcan U Küçükali C Tüzün E Çakir S. Erken Evre ve Kronik Bipolar Bozukluk Hastalarinda Inflamasyon ve Nörodejenerasyon Bulgulari. tur. [Inflammation and neurodegeneration in patients with early-stageand chronic bipolar disorder]. Turk Psikiyatri Derg. (2019) 30:75–81. Turkish. doi: 10.5080/u18376 31487372

[11] Berk M Dodd S Kauer-Sant'anna M Malhi GS Bourin M Kapczinski F . Dopamine dysregulation syndrome: implications for a dopamine hypothesis of bipolar disorder. Acta Psychiatr Scand Suppl. (2007) 116:41–9. doi: 10.1111/j.1600-0447.2007.01058.x 17688462

[12] Rosenblat JD McIntyre RS. Bipolar disorder and inflammation. Psychiatr Clin North Am. (2016) 39:125–37. doi: 10.1016/j.psc.2015.09.006 26876323

[13] Barbosa IG Bauer ME Machado-Vieira R Teixeira AL. Cytokines in bipolar disorder: paving the way for neuroprogression. Neural Plast. (2014) 2014:360481. doi: 10.1155/2014/360481 25313338 PMC4172873

[14] Bick RJ Poindexter BJ Kott MM Liang YA Dinh K Kaur B . Cytokines disrupt intracellular patterns of Parkinson's disease-associated proteins alpha-synuclein, tau and ubiquitin in cultured glial cells. Brain Res. (2008) 1217:203–12. doi: 10.1016/j.brainres.2008.03.081 18501880

[15] Page MJ Moher D Bossuyt PM Boutron I Hoffmann TC Mulrow CD . PRISMA 2020 explanation and elaboration: updated guidance and exemplars for reporting systematic reviews. BMJ. (2021) 372:n160. doi: 10.1136/bmj.n160 33781993 PMC8005925

[16] Liberati A Altman DG Tetzlaff J Mulrow C Gøtzsche PC Ioannidis JP . The PRISMA statement for reporting systematic reviews and meta-analyses of studies that evaluate healthcare interventions: explanation and elaboration. BMJ. (2009) 339:b2700. doi: 10.1136/bmj.b2700 19622552 PMC2714672

[17] Sterne JA Hernán MA Reeves BC Savović J Berkman ND Viswanathan M . ROBINS-I: a tool for assessing risk of bias in non-randomised studies of interventions. BMJ. (2016) 355:i4919. doi: 10.1136/bmj.i4919 27733354 PMC5062054

[18] Stang A. Critical evaluation of the Newcastle-Ottawa scale for the assessment of the quality of nonrandomized studies in meta-analyses. Eur J Epidemiol. (2010) 25:603–5. doi: 10.1007/s10654-010-9491-z 20652370

[19] Guyatt GH Oxman AD Vist GE Kunz R Falck-Ytter Y Alonso-Coello P . GRADE: an emerging consensus on rating quality of evidence and strength of recommendations. BMJ. (2008) 336:924–6. doi: 10.1136/bmj.39489.470347.AD 18436948 PMC2335261

[20] Higgins JP Thompson SG Deeks JJ Altman DG. Measuring inconsistency in meta-analyses. BMJ. (2003) 327:557–60. doi: 10.1136/bmj.327.7414.557 12958120 PMC192859

[21] Schandelmaier S Briel M Varadhan R Schmid CH Devasenapathy N Hayward RA . Development of the instrument to assess the credibility of effect modification analyses (ICEMAN) in randomized controlled trials and meta-analyses. CMAJ. (2020) 192:E901–6. doi: 10.1503/cmaj.200077 32778601 PMC7829020

[22] Nilsson FM Kessing LV Bolwig TG. Increased risk of developing Parkinson's disease for patients with major affective disorder: a register study. Acta Psychiatr Scand. (2001) 104:380–6. doi: 10.1111/j.1600-0447.2001.00372.x 11722320

[23] Lin HL Lin HC Chen YH. Psychiatric diseases predated the occurrence of Parkinson disease: a retrospective cohort study. Ann Epidemiol. (2014) 24:206–13. doi: 10.1016/j.annepidem.2013.12.010 24462274

[24] Marras C Herrmann N Fischer HD Fung K Gruneir A Rochon PA . Lithium use in older adults is associated with increased prescribing of Parkinson medications. Am J Geriatr Psychiatry. (2016) 24:301–9. doi: 10.1016/j.jagp.2015.11.004 27037047

[25] Huang MH Cheng CM Hsu JW Bai YM Su TP Li CT . Risk of subsequent Parkinson's disease among patients with bipolar disorder or major depression: a nationwide longitudinal study in Taiwan. Psychiatry Clin Neurosci. (2025) 79:29–36. doi: 10.1111/pcn.13759 39484734 PMC11693976

[26] Xu X Li Y Lu H Wang H Guo Y Dregan A . Prospective study of bipolar disorder and neurodegenerative diseases. NPJ Parkinsons Dis. (2024) 10:184. doi: 10.1038/s41531-024-00794-z 39362870 PMC11450157

[27] Yoon SY Lee SC Suh JH Yang SN Han K Kim YW. Different risks of early-onset and late-onset Parkinson disease in individuals with mental illness. NPJ Parkinsons Dis. (2024) 10:17. doi: 10.1038/s41531-023-00621-x 38195604 PMC10776668

[28] Shifman S Bronstein M Sternfeld M Pisanté A Weizman A Reznik I . COMT: a common susceptibility gene in bipolar disorder and schizophrenia. Am J Med Genet B Neuropsychiatr Genet. (2004) 128b:61–4. doi: 10.1002/ajmg.b.30032 15211633

[29] Abdolmaleky HM Cheng KH Faraone SV Wilcox M Glatt SJ Gao F . Hypomethylation of MB-COMT promoter is a major risk factor for schizophrenia and bipolar disorder. Hum Mol Genet. (2006) 15:3132–45. doi: 10.1093/hmg/ddl253 16984965 PMC2799943

[30] Zhang L Hu L Li X Zhang J Chen B. The DRD2 rs1800497 polymorphism increase the risk of mood disorder: evidence from an update meta-analysis. J Affect Disord. (2014) 158:71–7. doi: 10.1016/j.jad.2014.01.015 24655768

[31] Yu M Huang F Wang W Zhao C. Association between the DRD2 TaqIA gene polymorphism and Parkinson disease risk: an updated meta-analysis. Medicine (Baltimore). (2019) 98:e17136. doi: 10.1097/MD.0000000000017136 31517853 PMC6750301

[32] Dai D Wang Y Wang L Li J Ma Q Tao J . Polymorphisms of DRD2 and DRD3 genes and Parkinson's disease: a meta-analysis. Biomed Rep. (2014) 2:275–81. doi: 10.3892/br.2014.220 24649110 PMC3917740

[33] Soraya GV Ulhaq ZS Shodry S A'Raaf Sirojan Kusuma M Herawangsa S Sativa MO . Polymorphisms of the dopamine metabolic and signaling pathways are associated with susceptibility to motor levodopa-induced complications (MLIC) in Parkinson's disease: a systematic review and meta-analysis. Neurol Sci. (2022) 43:3649–70. doi: 10.1007/s10072-021-05829-4 35079903

[34] Tinsley RB Bye CR Parish CL Tziotis-Vais A George S Culvenor JG . Dopamine D2 receptor knockout mice develop features of Parkinson disease. Ann Neurol. (2009) 66:472–84. doi: 10.1002/ana.21716 19847912

[35] Modabbernia A Taslimi S Brietzke E Ashrafi M. Cytokine alterations in bipolar disorder: a meta-analysis of 30 studies. Biol Psychiatry. (2013) 74:15–25. doi: 10.1016/j.biopsych.2013.01.007 23419545

[36] Brietzke E Kauer-Sant'Anna M Teixeira AL Kapczinski F. Abnormalities in serum chemokine levels in euthymic patients with bipolar disorder. Brain Behav Immun. (2009) 23:1079–82. doi: 10.1016/j.bbi.2009.04.008 19406226

[37] Brietzke E Stertz L Fernandes BS Kauer-Sant'anna M Mascarenhas M Escosteguy Vargas A . Comparison of cytokine levels in depressed, manic and euthymic patients with bipolar disorder. J Affect Disord. (2009) 116:214–7. doi: 10.1016/j.jad.2008.12.001 19251324

[38] Breunis MN Kupka RW Nolen WA Suppes T Denicoff KD Leverich GS . High numbers of circulating activated T cells and raised levels of serum IL-2 receptor in bipolar disorder. Biol Psychiatry. (2003) 53:157–65. doi: 10.1016/S0006-3223(02)01452-X 12547472

[39] Naaldijk YM Bittencourt MC Sack U Ulrich H. Kinins and microglial responses in bipolar disorder: a neuroinflammation hypothesis. Biol Chem. (2016) 397:283–96. doi: 10.1515/hsz-2015-0257 26859499

[40] Jakobsson J Bjerke M Sahebi S Isgren A Ekman CJ Sellgren C . Monocyte and microglial activation in patients with mood-stabilized bipolar disorder. J Psychiatry Neurosci. (2015) 40:250–8. doi: 10.1503/jpn.140183 25768030 PMC4478058

[41] Nagatsu T Sawada M. Inflammatory process in Parkinson's disease: role for cytokines. Curr Pharm Des. (2005) 11:999–1016. doi: 10.2174/1381612053381620 15777250

[42] Kageyama Y Okura S Sukigara A Matsunaga A Maekubo K Oue T . The association among bipolar disorder, mitochondrial dysfunction, and reactive oxygen species. Biomolecules. (2025) 15:383. doi: 10.3390/biom15030383 40149919 PMC11940798

[43] Foubert-Samier A Helmer C Perez F Le Goff M Auriacombe S Elbaz A . Past exposure to neuroleptic drugs and risk of Parkinson disease in an elderly cohort. Neurology. (2012) 79:1615–21. doi: 10.1212/WNL.0b013e31826e25ce 23019267

[44] Ponzer K Millischer V Schalling M Gissler M Lavebratt C Backlund L. Lithium and risk of cardiovascular disease, dementia and venous thromboembolism. Bipolar Disord. (2023) 25:391–401. doi: 10.1111/bdi.13300 36651280

[45] Lei P Ayton S Appukuttan AT Moon S Duce JA Volitakis I . Lithium suppression of tau induces brain iron accumulation and neurodegeneration. Mol Psychiatry. (2017) 22:396–406. doi: 10.1038/mp.2016.96 27400857

[46] Chen Y Kang J Wu M Azuma A Zhao L. Differential association between HLA and diffuse panbronchiolitis in Northern and Southern Chinese. Intern Med. (2012) 51:271–6. doi: 10.2169/internalmedicine.51.6483 22293801

[47] Bond WS. Ethnicity and psychotropic drugs. Clin Pharm. (1991) 10:467–70. 1676623

[48] Liu B Dluzen DE. Oestrogen and nigrostriatal dopaminergic neurodegeneration: animal models and clinical reports of Parkinson's disease. Clin Exp Pharmacol Physiol. (2007) 34:555–65. doi: 10.1111/j.1440-1681.2007.04616.x 17581209

[49] Brann DW Dhandapani K Wakade C Mahesh VB Khan MM. Neurotrophic and neuroprotective actions of estrogen: basic mechanisms and clinical implications. Steroids. (2007) 72:381–405. doi: 10.1016/j.steroids.2007.02.003 17379265 PMC2048656

[50] Sawada H Shimohama S. Estrogens and Parkinson disease: novel approach for neuroprotection. Endocrine. (2003) 21:77–9. doi: 10.1385/ENDO:21:1:77 12777706

[51] Yi W Wu H Li R Li H Song Z She S . Prevalence and associated factors of obesity and overweight in Chinese patients with bipolar disorder. Front Psychiatry. (2022) 13:984829. doi: 10.3389/fpsyt.2022.984829 36147966 PMC9485538

[52] Chen J Guan Z Wang L Song G Ma B Wang Y. Meta-analysis: overweight, obesity, and Parkinson's disease. Int J Endocrinol. (2014) 2014:203930. doi: 10.1155/2014/203930 24672544 PMC3941583

[53] Chang KD Saxena K Howe M Simeonova D. Psychotropic medication exposure and age at onset of bipolar disorder in offspring of parents with bipolar disorder. J Child Adolesc Psychopharmacol. (2010) 20:25–32. doi: 10.1089/cap.2009.0036 20166793 PMC2835385

[54] Azami M Moradkhani A Afraie M Khateri S Sharifian E Zamani K . The risk of Parkinson's disease in diabetic people: an updated systematic review and meta-analysis. Acta Neurol Belg. (2024) 124:775–90. doi: 10.1007/s13760-023-02424-6 37982931

[55] Tanner CM Kamel F Ross GW Hoppin JA Goldman SM Korell M . Rotenone, paraquat, and Parkinson's disease. Environ Health Perspect. (2011) 119:866–72. doi: 10.1289/ehp.1002839 21269927 PMC3114824

[56] Kmiecik MJ Micheletti S Coker D Heilbron K Shi J Stagaman K . Genetic analysis and natural history of Parkinson's disease due to the LRRK2 G2019S variant. Brain. (2024) 147:1996–2008. doi: 10.1093/brain/awae073 38804604 PMC11146432

