# Supplementary figures and images for: Bipolar disorder and subsequent Parkinson's disease: a meta-analysis of cohort studies
Source: Front Neurol. 2026 Jun 5;17:1825046. doi: 10.3389/fneur.2026.1825046 (PMC13278865; doi:10.3389/fneur.2026.1825046)

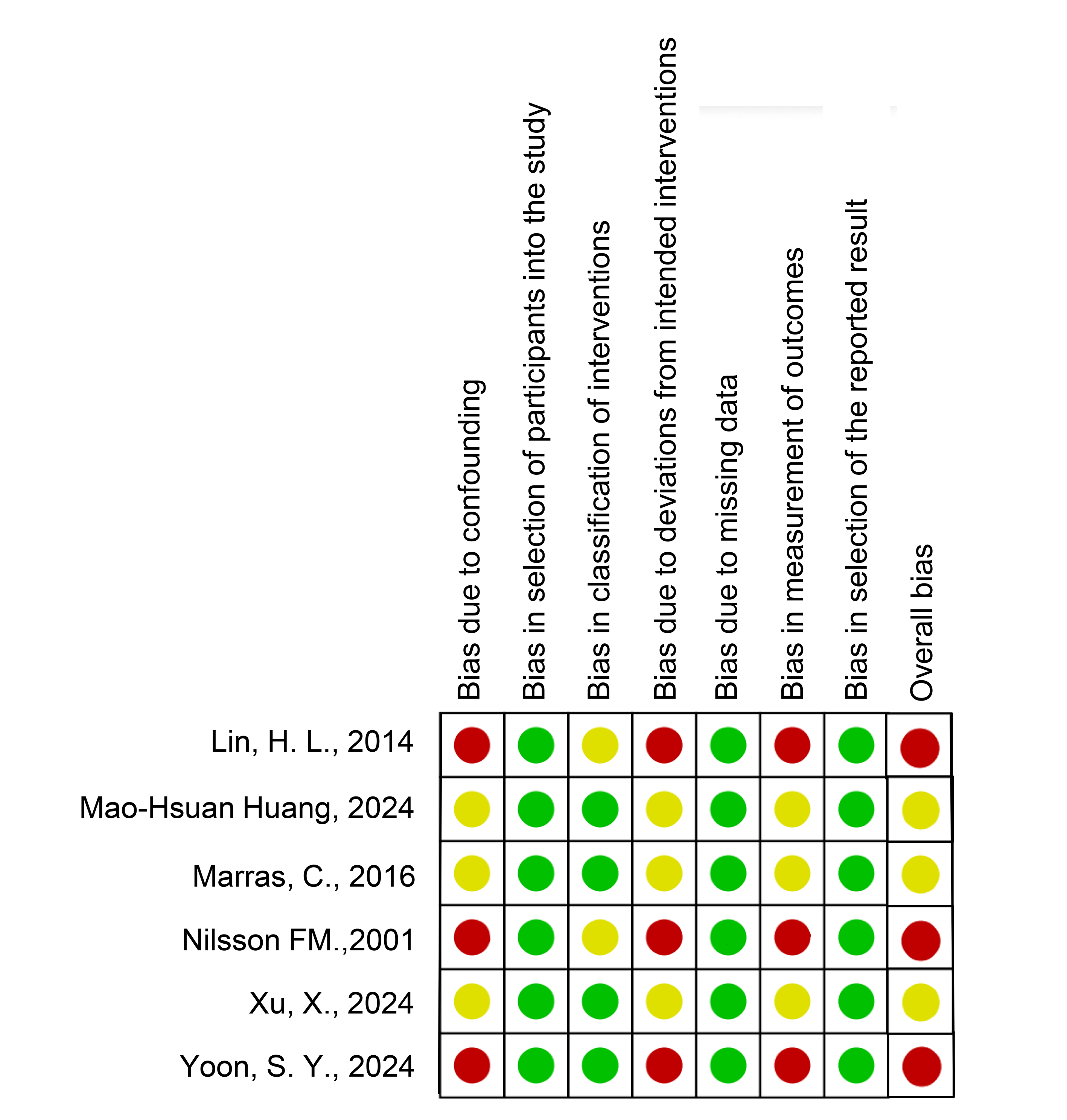

Supplement: Supplementary Figure 1 — ROBINS-I traffic-light plot of risk of bias across seven domains for the six included studies. Red, serious; yellow, moderate; green, low. [file Image_1.TIF]

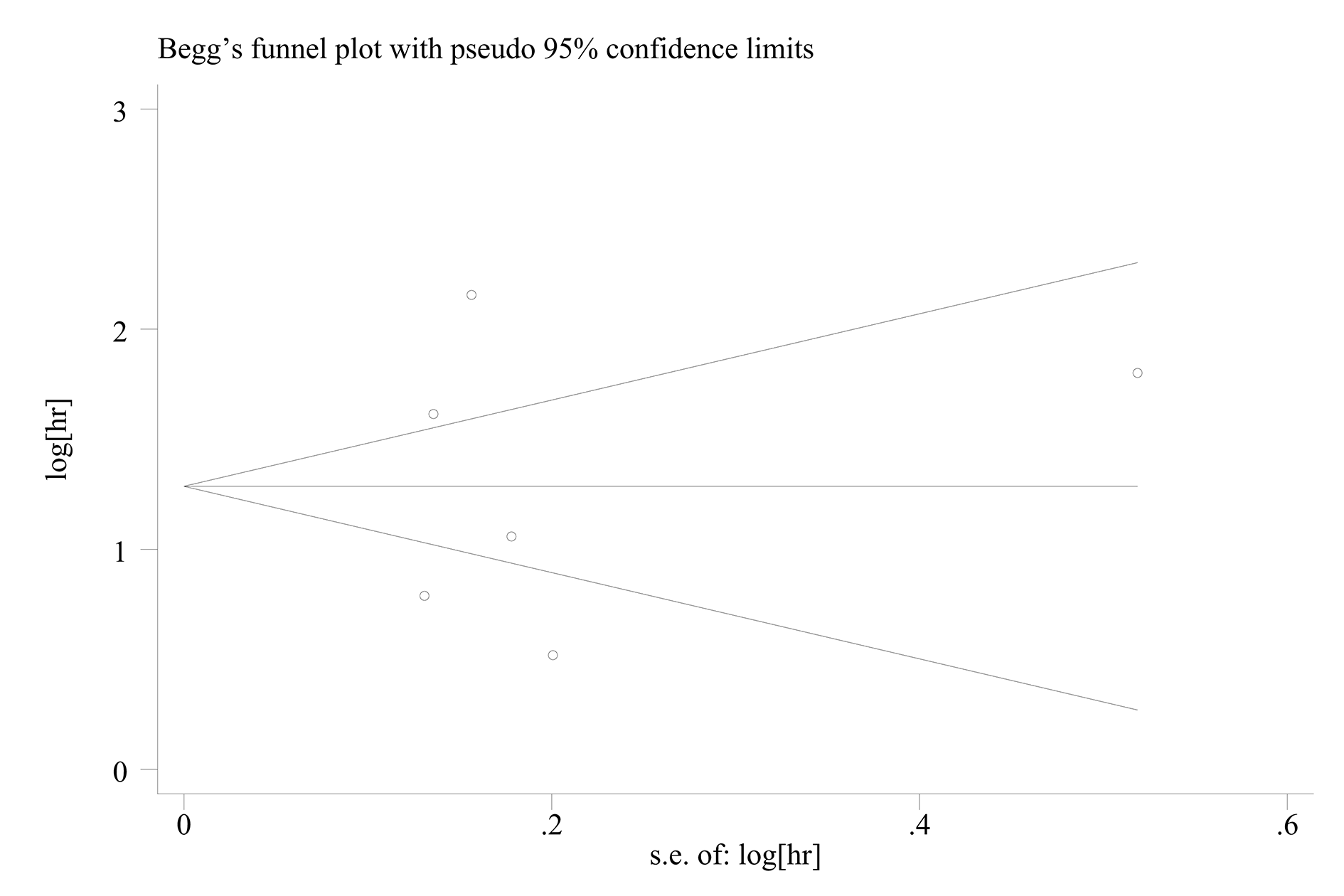

Supplement: Supplementary Figure 2 — Funnel plot of the six included studies. With fewer than 10 studies, statistical power to detect asymmetry is insufficient; this plot is provided for descriptive purposes only and should not be interpreted as evidence of publication bias. [file Image_2.TIF]

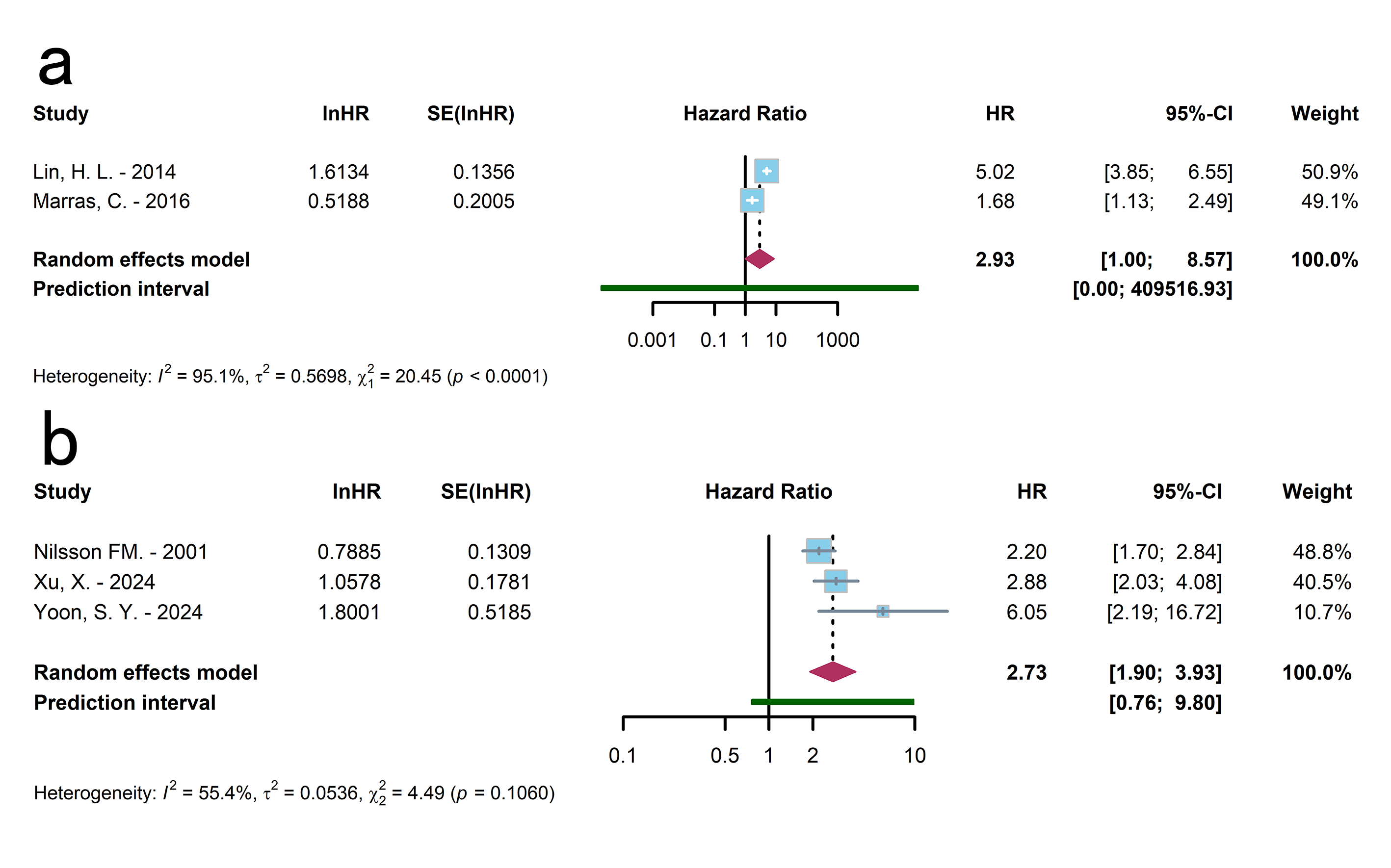

Supplement: Supplementary Figure 3 — Forest plot of subgroup analysis of PD incidence in BD patients based on follow-up time (a: < 7 years: P = 0.049; b: >7 years: P < 0.001). [file Image_3.TIF]
