# Supplementary material for: Bipolar disorder and subsequent Parkinson's disease: a meta-analysis of cohort studies
Source: Front Neurol. 2026 Jun 5;17:1825046. doi: 10.3389/fneur.2026.1825046 (PMC13278865; doi:10.3389/fneur.2026.1825046)
Supplement: Supplementary Table 1 — Characteristics of the studies included in the meta-analysis. [file Table_1.docx]

**Supplementary Table 1. Characteristics of included studies in the meta-analysis.**

| Author, Year | Exclusion criteria | Exposure group | Control group | Age (year) | Female % | Adjusted factors | Number | | | |
| --- | --- | --- | --- | --- | --- | --- | --- | --- | --- | --- |
|  |  |  |  |  |  |  | BD group | Control group | BD with PD | Control with PD |
| Nilsson FM. ,2001 | Patients with diagnosis of neurological disorder, osteoarthritis or diabetes as an auxiliary diagnosis at the first charge and a previous diagnosis of neurological disorder, osteoarthritis or diabetes as a main or auxiliary diagnosis in the “the reverse register” | Affective episode | Osteoarthritis | NA | NA | Age, gender | 2007 | 81380 | NA | NA |
| Lin, H. L., 2014 | Age<18 years and patients with a PD diagnosis before index date | BD | Comparison | NA | 38.4 | Age, gender, income, level of urbanization, geographical location, dyslipidemia, diabetes, hypertension, coronary heart disease, obesity, alcohol | 1203 | 220791 | 62 | 2155 |
| Mao-Hsuan Huang, 2024 | Patients with PD and related diseases; patients with previous diagnoses of major psychiatric disorders or PD | BD | Control | 45-65 | 59.1 | Age, gender, income, level of urbanization, clinical visits, Charlson score, smoking, medical comorbidities | 21186 | 42374 | 300 | 50 |
| Marras, C., 2016 | Age>105 years; a history of treatment with medication of Parkinson’s disease; have a history of the following in the 5 years prior to index date; a diagnostic code of Parkinson's disease or Parkinsonism (OHIP 332, ICD9: 332 or 333, ICD10: G20, G22, G211,G212, G213, G214, G218, G219, F023); with diagnostic code for epilepsy or a palliative care code in the 1 year prior to index data | Lithium | Antidepressant | NA | 54.0 to 58.0 | Age, long term care residence, dementia, Charlson score, antipsychotic use after index date | 1749 | 285154 | 51 | 4594 |
| Xu, X., 2024 | Withdraw from UK Biobank; Participants with prevalent dementia or Parkinson disease | BD | Without BD | 56.50 | 54.4 | Age, gender, ethnicity, BMI, smoking, educational level, alcohol, physical activity, coffee intake, history of cancer, CVD, diabetes, hypertension, BD-PRS | 2250 | 498983 | 32 | 3425 |
| Yoon, S. Y., 2024 | Missing data; PD diagnosis before enrollment; PD with 1-year lag period | BD | Control | <50years, 37.25±7.63  >50years, 60.67±8.33 | <50years, 40.8  >50years, 51.6 | Age, gender, alcohol, physical activity, income, BMI, diabetes, hypertension, dyslipidemia | 16319 | 8850972 | NA | NA |

BMI: Body mass index; CVD: Cardiovascular disease; BD: Bipolar disorder; ICD: International Statistical Classification of Diseases and Related Health Problems; BD-PRS: Bipolar Disorder- Polygenic Risk Score; NA: Not available; PD: Parkinson’s disease; OHIP: Ontario Health Insurance Plan; UK: United Kingdom
