# Supplementary material for: Bipolar disorder and subsequent Parkinson's disease: a meta-analysis of cohort studies
Source: Front Neurol. 2026 Jun 5;17:1825046. doi: 10.3389/fneur.2026.1825046 (PMC13278865; doi:10.3389/fneur.2026.1825046)
Supplement: Supplementary Table 2 — Risk of bias assessment of ROBINS-I (risk of bias judgements in non-randomized studies of interventions). [file Table_2.docx]

| Author, year | Bias due to confounding | Bias in selection of participants into the study | Bias in classification of interventions | Bias due to deviations from intended interventions | Bias due to missing data | Bias in measurement of outcomes | Bias in selection of the reported result | Overall bias |
| --- | --- | --- | --- | --- | --- | --- | --- | --- |
| Nilsson FM.,2001 | Serious | Low | Moderate | Serious | Low | Serious | Low | Serious |
| Lin, H. L., 2014 | Serious | Low | Moderate | Serious | Low | Serious | Low | Serious |
| Mao-Hsuan Huang, 2024 | Moderate | Low | Low | Moderate | Low | Moderate | Low | Moderate |
| Marras, C., 2016 | Moderate | Low | Low | Moderate | Low | Moderate | Low | Moderate |
| Xu, X., 2024 | Moderate | Low | Low | Moderate | Low | Moderate | Low | Moderate |
| Yoon, S. Y., 2024 | Serious | Low | Low | Serious | Low | Serious | Low | Serious |

**Supplementary Table2. Risk of bias assessment of ROBINS-I (risk of bias judgements in non-randomized studies of interventions)**

ROBINS-I: Risk Of Bias In Non-randomized Studies of Interventions
