# Supplementary material for: Bipolar disorder and subsequent Parkinson's disease: a meta-analysis of cohort studies
Source: Front Neurol. 2026 Jun 5;17:1825046. doi: 10.3389/fneur.2026.1825046 (PMC13278865; doi:10.3389/fneur.2026.1825046)
Supplement: Supplementary Table 3 — Quality assessment of cohort studies included. [file Table_3.doc]

**Supplementary Table 3. Quality assessment of cohort studies included.**

| Author, year | **Selection (Out of 4)** | | | | **Comparability**  **(Out of 2)** | **Outcomes (Out of 3)** | | | **Total**  **(Out of 9)** |
| --- | --- | --- | --- | --- | --- | --- | --- | --- | --- |
|  | Representativeness of exposed cohort | Selection of non exposed cohort | Ascertainment  of exposure | Outcome not present at the start of the study |  | Assessment of outcomes | Length of follow-up | Adequacy of follow up of cohorts |  |
| Nilsson FM. ,2001 | 1 | 1 | 1 | 1 | 1 | 1 | 1 | 1 | 8 |
| Lin, H. L. , 2014 | 1 | 1 | 1 | 1 | 1 | 1 | 1 | 0 | 7 |
| Mao-Hsuan Huang, 2024 | 1 | 1 | 1 | 1 | 2 | 1 | 1 | 0 | 8 |
| Marras, C. , 2016 | 1 | 1 | 1 | 1 | 1 | 1 | 1 | 0 | 7 |
| Xu, X. , 2024 | 1 | 1 | 1 | 1 | 1 | 1 | 1 | 1 | 8 |
| Yoon, S. Y. , 2024 | 1 | 1 | 1 | 1 | 2 | 1 | 1 | 1 | 9 |

The cohort studies were assessed by the Newcastle-Ottawa Quality Assessment Scale (NOS) checklist.
