# Supplementary material for: Bipolar disorder and subsequent Parkinson's disease: a meta-analysis of cohort studies
Source: Front Neurol. 2026 Jun 5;17:1825046. doi: 10.3389/fneur.2026.1825046 (PMC13278865; doi:10.3389/fneur.2026.1825046)
Supplement: Supplementary Table 4 — ICEMAN assessment of the credibility of subgroup analyses. [file Table_4.docx]

| Subgroup | Is the analysis of effect modification based on comparison within rather than between trials? | For within-trial comparisons, is the effect modification similar from trial to trial? | For between-trial comparisons, is the number of trials large? | Was the direction of effect modification correctly hypothesized a priori? | Does a test for interaction suggest that chance is an unlikely explanation of the apparent effect modification? | Did the authors test only a small number of effect modifiers or consider the number in their statistical analysis? | Did the authors use a random effects model? | If the effect modifier is a continuous variable, were arbitrary cut points avoided? | Overall credibility |
| --- | --- | --- | --- | --- | --- | --- | --- | --- | --- |
| Sex | Mostly between or unclear | Probably not similar or unclear | Very small | Definitely no | Chance a very likely explanation | Probably no or unclear | Definitely yes | Not applicable | Very low |
| Age at onset (<50 vs ≥50) | Mostly between or unclear | Probably not similar or unclear | Very small | Probably yes | Chance a very likely explanation | Probably no or unclear | Definitely yes | Probably no or unclear | Very low |
| Geographic region (Eastern vs Western) | Completely between | Not applicable | Rather small or unclear | Probably yes | Chance an unlikely explanation | Probably no or unclear | Definitely yes | Not applicable | Very low |
| Follow-up duration | Completely between | Not applicable | Very small | Definitely no | Chance a very likely explanation | Probably no or unclear | Definitely yes | Definitely no | Very low |
| NOS score | Completely between | Not applicable | Very small | Definitely no | Chance a very likely explanation | Probably no or unclear | Definitely yes | Definitely no | Very low |

**Supplementary Table 4. ICEMAN assessment of the credibility of subgroup analyses**

ICEMAN：Instrument for assessing the Credibility of Effect Modification Analyses; NOS: Newcastle-Ottawa Quality Assessment Scale
