# Supplementary material for: Bipolar disorder and subsequent Parkinson's disease: a meta-analysis of cohort studies
Source: Front Neurol. 2026 Jun 5;17:1825046. doi: 10.3389/fneur.2026.1825046 (PMC13278865; doi:10.3389/fneur.2026.1825046)
Supplement: Supplementary Table 5 — GRADE summary of findings: certainty of evidence for the association between bipolar disorder and subsequent Parkinson's disease diagnosis. [file Table_5.docx]

**Author(s):**

**Question:** Bipolar disorder compared to Control for PD incidence **Setting:**

**Bibliography:**

| **Certainty assessment** | | | | | | | **№ of patients** | | **Effect** | | **Certainty** | **Importance** |
| --- | --- | --- | --- | --- | --- | --- | --- | --- | --- | --- | --- | --- |
| **№ of studies** | **Study design** | **Risk of bias** | **Inconsistency** | **Indirectness** | **Imprecision** | **Other considerations** | **bipolar disorder** | **Control** | **Relative (95% CI)** | **Absolute (95% CI)** |  |  |

**The incidence of PD (follow-up: range 6 years to 9 years; assessed with: Hospital discharge diagnosis and/or antiparkinsonian drug dispensing)**

| 6 | non-  randomised  studies | seriousa | seriousb | not serious | seriousc | none | 44714  participants | 9979654  participants | **HR 3.65**  (2. 16 to 6. 17)  [The incidence of  PD] | **-- per 1,000**  (from -- to --) | ⨁◯◯◯  Very low a, b, c | CRITICAL |
| --- | --- | --- | --- | --- | --- | --- | --- | --- | --- | --- | --- | --- |
|  |  |  |  |  |  |  | - | 0 .0% |  | **-- per 1,000**  (from -- to --) |  |  |

**CI:** confidence interval; **HR:** hazard ratio

**Explanations**

a. Three of six included cohort studies were judged to be at serious overall risk of bias using ROBINS -I. Critical concerns related to confounding (Domain 1) and measurement of outcomes (Domain 6): four studies did not adequately exclude or adjust for exposure to antipsychotics, lithium, or valproate—medications well-documented to cause drug-induced parkinsonism that is clinically indistinguishable from idiopathic Parkinson 's disease in administrative databases; three studies relied solely on diagnostic codes without neurologist validation or medical record review, precluding reliable distinction between drug-induced parkinsonism and idiopathic Parkinson 's disease.

b. Statistical heterogeneity was extreme (I² > 90%, τ² substantial), and the 95% prediction interval was wide, indicating that the true effect may vary substantially across settings or even include the null. Pooled subgroup hazard ratios ranged from 1.70 to

10.56 depending on age at onset, sex, and region, further suggesting that a single common underlying effect is unlikely. With only six studies, meta-regression to explore sources of heterogeneity was not feasible.

c. The optimal information size was not met for a reliable effect estimate. Although the total number of participants was large, the number of Parkinson's disease events was relatively small in several subgroups (e.g., early-onset Parkinson’ s disease, sex-

stratified analyses). The 95% confidence interval for the pooled hazard ratio was wide and included values that would imply both moderate and very large clinical effects. Additionally, subgroup analyses were based on only 2–3 studies with very few events, producing extremely imprecise estimates. Consequently, the confidence intervals are consistent with conflicting clinical decisions.
