# Supplementary material for: Bipolar disorder and subsequent Parkinson's disease: a meta-analysis of cohort studies
Source: Front Neurol. 2026 Jun 5;17:1825046. doi: 10.3389/fneur.2026.1825046 (PMC13278865; doi:10.3389/fneur.2026.1825046)
Supplement: Supplementary Search Strategy — Detailed search strategy for PubMed, web of science, Embase and Cochrane library. [file Table_6.docx]

**Supplementary** **Search Strategy.**

**Pubmed**

| Search number | Query | Search Details | Results |
| --- | --- | --- | --- |
| 1 | ((((((((((("Parkinson Disease"[Mesh]) OR (Idiopathic Parkinson Disease)) OR (Idiopathic Parkinson's Disease)) OR (Lewy Body Parkinson Disease)) OR (Lewy Body Parkinson's Disease)) OR (Paralysis Agitans)) OR (Parkinson Disease, Idiopathic)) OR (Parkinson's Disease)) OR (Parkinson's Disease, Idiopathic)) OR (Parkinson's Disease, Lewy Body)) OR (Primary Parkinsonism)) OR (Parkinsonism, Primary) | "Parkinson Disease"[MeSH Terms] OR ("Parkinson Disease"[MeSH Terms] OR ("parkinson"[All Fields] AND "disease"[All Fields]) OR "Parkinson Disease"[All Fields] OR ("idiopathic"[All Fields] AND "parkinson"[All Fields] AND "disease"[All Fields]) OR "idiopathic parkinson disease"[All Fields]) OR ("Parkinson Disease"[MeSH Terms] OR ("parkinson"[All Fields] AND "disease"[All Fields]) OR "Parkinson Disease"[All Fields] OR ("idiopathic"[All Fields] AND "parkinson s"[All Fields] AND "disease"[All Fields]) OR "idiopathic parkinson s disease"[All Fields]) OR ("Parkinson Disease"[MeSH Terms] OR ("parkinson"[All Fields] AND "disease"[All Fields]) OR "Parkinson Disease"[All Fields] OR ("lewy"[All Fields] AND "body"[All Fields] AND "parkinson"[All Fields] AND "disease"[All Fields]) OR "lewy body parkinson disease"[All Fields]) OR ("Parkinson Disease"[MeSH Terms] OR ("parkinson"[All Fields] AND "disease"[All Fields]) OR "Parkinson Disease"[All Fields] OR ("lewy"[All Fields] AND "body"[All Fields] AND "parkinson s"[All Fields] AND "disease"[All Fields]) OR "lewy body parkinson s disease"[All Fields]) OR ("Parkinson Disease"[MeSH Terms] OR ("parkinson"[All Fields] AND "disease"[All Fields]) OR "Parkinson Disease"[All Fields] OR ("paralysis"[All Fields] AND "agitans"[All Fields]) OR "paralysis agitans"[All Fields]) OR ("Parkinson Disease"[MeSH Terms] OR ("parkinson"[All Fields] AND "disease"[All Fields]) OR "Parkinson Disease"[All Fields] OR ("parkinson"[All Fields] AND "disease"[All Fields] AND "idiopathic"[All Fields]) OR "parkinson disease idiopathic"[All Fields]) OR ("Parkinson Disease"[MeSH Terms] OR ("parkinson"[All Fields] AND "disease"[All Fields]) OR "Parkinson Disease"[All Fields] OR ("parkinson s"[All Fields] AND "disease"[All Fields]) OR "parkinson s disease"[All Fields]) OR ("Parkinson Disease"[MeSH Terms] OR ("parkinson"[All Fields] AND "disease"[All Fields]) OR "Parkinson Disease"[All Fields] OR ("parkinson s"[All Fields] AND "disease"[All Fields] AND "idiopathic"[All Fields]) OR "parkinson s disease idiopathic"[All Fields]) OR ("Parkinson Disease"[MeSH Terms] OR ("parkinson"[All Fields] AND "disease"[All Fields]) OR "Parkinson Disease"[All Fields] OR ("parkinson s"[All Fields] AND "disease"[All Fields] AND "lewy"[All Fields] AND "body"[All Fields]) OR "parkinson s disease lewy body"[All Fields]) OR ("Parkinson Disease"[MeSH Terms] OR ("parkinson"[All Fields] AND "disease"[All Fields]) OR "Parkinson Disease"[All Fields] OR ("primary"[All Fields] AND "parkinsonism"[All Fields]) OR "primary parkinsonism"[All Fields]) OR ("Parkinson Disease"[MeSH Terms] OR ("parkinson"[All Fields] AND "disease"[All Fields]) OR "Parkinson Disease"[All Fields] OR ("parkinsonism"[All Fields] AND "primary"[All Fields]) OR "parkinsonism primary"[All Fields]) | 171209 |
| 2 | (((((((((((((((((((((((((((("Bipolar Disorder"[Mesh]) OR (Bipolar Disorders)) OR (Disorder, Bipolar)) OR (Bipolar Mood Disorder)) OR (Bipolar Mood Disorders)) OR (Disorder, Bipolar Mood)) OR (Mood Disorder, Bipolar)) OR (Manic Depression)) OR (Depression, Manic)) OR (Depressions, Manic)) OR (Affective Psychosis, Bipolar)) OR (Bipolar Affective Psychosis)) OR (Psychoses, Bipolar Affective)) OR (Psychosis, Bipolar Affective)) OR (Psychoses, Manic-Depressive)) OR (Psychoses, Manic Depressive)) OR (Manic-Depressive Psychosis)) OR (Manic Depressive Psychosis)) OR (Psychosis, Manic-Depressive)) OR (Psychosis, Manic Depressive)) OR (Depression, Bipolar)) OR (Bipolar Depression)) OR (Manic Disorder)) OR (Disorder, Manic)) OR (Manic Disorders)) OR (Bipolar Disorder Type 1)) OR (Type 1 Bipolar Disorder)) OR (Bipolar Disorder Type 2)) OR (Type 2 Bipolar Disorder) | "Bipolar Disorder"[MeSH Terms] OR ("Bipolar Disorder"[MeSH Terms] OR ("bipolar"[All Fields] AND "disorder"[All Fields]) OR "Bipolar Disorder"[All Fields] OR ("bipolar"[All Fields] AND "disorders"[All Fields]) OR "bipolar disorders"[All Fields]) OR ("Bipolar Disorder"[MeSH Terms] OR ("bipolar"[All Fields] AND "disorder"[All Fields]) OR "Bipolar Disorder"[All Fields] OR ("disorder"[All Fields] AND "bipolar"[All Fields]) OR "disorder bipolar"[All Fields]) OR ("Bipolar Disorder"[MeSH Terms] OR ("bipolar"[All Fields] AND "disorder"[All Fields]) OR "Bipolar Disorder"[All Fields] OR ("bipolar"[All Fields] AND "mood"[All Fields] AND "disorder"[All Fields]) OR "bipolar mood disorder"[All Fields]) OR ("Bipolar Disorder"[MeSH Terms] OR ("bipolar"[All Fields] AND "disorder"[All Fields]) OR "Bipolar Disorder"[All Fields] OR ("bipolar"[All Fields] AND "mood"[All Fields] AND "disorders"[All Fields]) OR "bipolar mood disorders"[All Fields]) OR ("Bipolar Disorder"[MeSH Terms] OR ("bipolar"[All Fields] AND "disorder"[All Fields]) OR "Bipolar Disorder"[All Fields] OR ("disorder"[All Fields] AND "bipolar"[All Fields] AND "mood"[All Fields]) OR "disorder bipolar mood"[All Fields]) OR ("Bipolar Disorder"[MeSH Terms] OR ("bipolar"[All Fields] AND "disorder"[All Fields]) OR "Bipolar Disorder"[All Fields] OR ("mood"[All Fields] AND "disorder"[All Fields] AND "bipolar"[All Fields]) OR "mood disorder bipolar"[All Fields]) OR ("Bipolar Disorder"[MeSH Terms] OR ("bipolar"[All Fields] AND "disorder"[All Fields]) OR "Bipolar Disorder"[All Fields] OR ("manic"[All Fields] AND "depression"[All Fields]) OR "manic depression"[All Fields]) OR ("Bipolar Disorder"[MeSH Terms] OR ("bipolar"[All Fields] AND "disorder"[All Fields]) OR "Bipolar Disorder"[All Fields] OR ("depression"[All Fields] AND "manic"[All Fields]) OR "depression manic"[All Fields]) OR ("Bipolar Disorder"[MeSH Terms] OR ("bipolar"[All Fields] AND "disorder"[All Fields]) OR "Bipolar Disorder"[All Fields] OR ("depressions"[All Fields] AND "manic"[All Fields]) OR "depressions manic"[All Fields]) OR ("Bipolar Disorder"[MeSH Terms] OR ("bipolar"[All Fields] AND "disorder"[All Fields]) OR "Bipolar Disorder"[All Fields] OR ("affective"[All Fields] AND "psychosis"[All Fields] AND "bipolar"[All Fields]) OR "affective psychosis bipolar"[All Fields]) OR ("Bipolar Disorder"[MeSH Terms] OR ("bipolar"[All Fields] AND "disorder"[All Fields]) OR "Bipolar Disorder"[All Fields] OR ("bipolar"[All Fields] AND "affective"[All Fields] AND "psychosis"[All Fields]) OR "bipolar affective psychosis"[All Fields]) OR ("Bipolar Disorder"[MeSH Terms] OR ("bipolar"[All Fields] AND "disorder"[All Fields]) OR "Bipolar Disorder"[All Fields] OR ("psychoses"[All Fields] AND "bipolar"[All Fields] AND "affective"[All Fields]) OR "psychoses bipolar affective"[All Fields]) OR ("Bipolar Disorder"[MeSH Terms] OR ("bipolar"[All Fields] AND "disorder"[All Fields]) OR "Bipolar Disorder"[All Fields] OR ("psychosis"[All Fields] AND "bipolar"[All Fields] AND "affective"[All Fields]) OR "psychosis bipolar affective"[All Fields]) OR ("Bipolar Disorder"[MeSH Terms] OR ("bipolar"[All Fields] AND "disorder"[All Fields]) OR "Bipolar Disorder"[All Fields] OR ("psychoses"[All Fields] AND "manic"[All Fields] AND "depressive"[All Fields]) OR "psychoses manic depressive"[All Fields]) OR ("Bipolar Disorder"[MeSH Terms] OR ("bipolar"[All Fields] AND "disorder"[All Fields]) OR "Bipolar Disorder"[All Fields] OR ("psychoses"[All Fields] AND "manic"[All Fields] AND "depressive"[All Fields]) OR "psychoses manic depressive"[All Fields]) OR ("Bipolar Disorder"[MeSH Terms] OR ("bipolar"[All Fields] AND "disorder"[All Fields]) OR "Bipolar Disorder"[All Fields] OR ("manic"[All Fields] AND "depressive"[All Fields] AND "psychosis"[All Fields]) OR "manic depressive psychosis"[All Fields]) OR ("Bipolar Disorder"[MeSH Terms] OR ("bipolar"[All Fields] AND "disorder"[All Fields]) OR "Bipolar Disorder"[All Fields] OR ("manic"[All Fields] AND "depressive"[All Fields] AND "psychosis"[All Fields]) OR "manic depressive psychosis"[All Fields]) OR ("Bipolar Disorder"[MeSH Terms] OR ("bipolar"[All Fields] AND "disorder"[All Fields]) OR "Bipolar Disorder"[All Fields] OR ("psychosis"[All Fields] AND "manic"[All Fields] AND "depressive"[All Fields]) OR "psychosis manic depressive"[All Fields]) OR ("Bipolar Disorder"[MeSH Terms] OR ("bipolar"[All Fields] AND "disorder"[All Fields]) OR "Bipolar Disorder"[All Fields] OR ("psychosis"[All Fields] AND "manic"[All Fields] AND "depressive"[All Fields]) OR "psychosis manic depressive"[All Fields]) OR ("Bipolar Disorder"[MeSH Terms] OR ("bipolar"[All Fields] AND "disorder"[All Fields]) OR "Bipolar Disorder"[All Fields] OR ("depression"[All Fields] AND "bipolar"[All Fields]) OR "depression bipolar"[All Fields]) OR ("Bipolar Disorder"[MeSH Terms] OR ("bipolar"[All Fields] AND "disorder"[All Fields]) OR "Bipolar Disorder"[All Fields] OR ("bipolar"[All Fields] AND "depression"[All Fields]) OR "bipolar depression"[All Fields]) OR ("Bipolar Disorder"[MeSH Terms] OR ("bipolar"[All Fields] AND "disorder"[All Fields]) OR "Bipolar Disorder"[All Fields] OR ("manic"[All Fields] AND "disorder"[All Fields]) OR "manic disorder"[All Fields]) OR ("Bipolar Disorder"[MeSH Terms] OR ("bipolar"[All Fields] AND "disorder"[All Fields]) OR "Bipolar Disorder"[All Fields] OR ("disorder"[All Fields] AND "manic"[All Fields]) OR "disorder manic"[All Fields]) OR ("Bipolar Disorder"[MeSH Terms] OR ("bipolar"[All Fields] AND "disorder"[All Fields]) OR "Bipolar Disorder"[All Fields] OR ("manic"[All Fields] AND "disorders"[All Fields]) OR "manic disorders"[All Fields]) OR ("Bipolar Disorder"[MeSH Terms] OR ("bipolar"[All Fields] AND "disorder"[All Fields]) OR "Bipolar Disorder"[All Fields] OR "bipolar disorder type 1"[All Fields]) OR ("Bipolar Disorder"[MeSH Terms] OR ("bipolar"[All Fields] AND "disorder"[All Fields]) OR "Bipolar Disorder"[All Fields] OR "type 1 bipolar disorder"[All Fields]) OR ("Bipolar Disorder"[MeSH Terms] OR ("bipolar"[All Fields] AND "disorder"[All Fields]) OR "Bipolar Disorder"[All Fields] OR "bipolar disorder type 2"[All Fields]) OR ("Bipolar Disorder"[MeSH Terms] OR ("bipolar"[All Fields] AND "disorder"[All Fields]) OR "Bipolar Disorder"[All Fields] OR "type 2 bipolar disorder"[All Fields]) | 73532 |
| 3 | #1 AND #2 | #1 AND #2 | 755 |

**Web of Science**

| Search number | Query | Search Details | Results |
| --- | --- | --- | --- |
| 1 | (((((((((((ALL=(Parkinson disease)) OR ALL=(Idiopathic Parkinson Disease)) OR ALL=(Idiopathic Parkinson's Disease)) OR ALL=(Lewy Body Parkinson Disease)) OR ALL=(Lewy Body Parkinson's Disease)) OR ALL=(Paralysis Agitans)) OR ALL=(Parkinson Disease, Idiopathic)) OR ALL=(Parkinson's Disease)) OR ALL=(Parkinson's Disease, Idiopathic)) OR ALL=(Parkinson's Disease, Lewy Body)) OR ALL=(Primary Parkinsonism)) OR ALL=(Parkinsonism, Primary) | Parkinson disease (All Fields) or Idiopathic Parkinson Disease (All Fields) or Idiopathic Parkinson's Disease (All Fields) or Lewy Body Parkinson Disease (All Fields) or Lewy Body Parkinson's Disease (All Fields) or Paralysis agitate (All Fields) or Parkinson Disease, Idiopathic (All Fields) or Parkinson's Disease (All Fields) or Parkinson's Disease, Idiopathic (All Fields) or Parkinson's Disease, Lewy Body (All Fields) or Primary Parkinsonism (All Fields) or Parkinsonism, Primary (All Fields) | 157083 |
| 2 | ((((((((((((((((((((((((((((ALL=(Bipolar Disorder)) OR ALL=(Bipolar Disorders)) OR ALL=(Disorder, Bipolar)) OR ALL=(Bipolar Mood Disorder)) OR ALL=(Bipolar Mood Disorders)) OR ALL=(Disorder, Bipolar Mood)) OR ALL=(Mood Disorder, Bipolar)) OR ALL=(Manic Depression)) OR ALL=(Depression, Manic)) OR ALL=(Depressions, Manic)) OR ALL=(Affective Psychosis, Bipolar)) OR ALL=(Bipolar Affective Psychosis)) OR ALL=(Psychoses, Bipolar Affective)) OR ALL=(Psychosis, Bipolar Affective)) OR ALL=(Psychoses, Manic-Depressive)) OR ALL=(Psychoses, Manic Depressive)) OR ALL=(Manic-Depressive Psychosis)) OR ALL=(Manic Depressive Psychosis)) OR ALL=(Psychosis, Manic-Depressive)) OR ALL=(Psychosis, Manic Depressive)) OR ALL=(Depression, Bipolar)) OR ALL=(Bipolar Depression)) OR ALL=(Manic Disorder)) OR ALL=(Disorder, Manic)) AND ALL=(Manic Disorders )) OR ALL=(Bipolar Disorder Type 1)) OR ALL=(Type 1 Bipolar Disorder)) OR ALL=(Bipolar Disorder Type 2)) OR ALL=(Type 2 Bipolar Disorder ) | Bipolar Disorder (All Fields) OR Bipolar Disorders (All Fields) OR Disorder, Bipolar (All Fields) OR Bipolar Mood Disorder (All Fields) OR Bipolar Mood Disorders (All Fields) OR Disorder, Bipolar Mood (All Fields) OR Mood Disorder, Bipolar (All Fields) OR Manic Depression (All Fields) OR Depression, Manic (All Fields) OR Depressions, Manic (All Fields) OR Affective Psychosis, Bipolar (All Fields) OR Bipolar Affective Psychosis (All Fields) OR Psychoses, Bipolar Affective (All Fields) OR Psychosis, Bipolar Affective (All Fields) OR Psychoses, Manic-Depressive (All Fields) OR Psychoses, Manic Depressive (All Fields) OR Manic-Depressive Psychosis (All Fields) OR Manic Depressive Psychosis (All Fields) OR Psychosis, Manic-Depressive (All Fields) OR Psychosis, Manic Depressive (All Fields) OR Depression, Bipolar (All Fields) OR Bipolar Depression (All Fields) OR Manic Disorder (All Fields) OR Disorder, Manic (All Fields) OR Manic Disorders (All Fields) OR Bipolar Disorder Type 1 (All Fields) OR Type 1 Bipolar Disorder (All Fields) OR Bipolar Disorder Type 2 (All Fields) OR Type 2 Bipolar Disorder (All Fields) | 62731 |
| 3 | #1 AND #2 | #1 AND #2 | 627 |

**EMBASE**

| Search number | Query | Search Details | Results |
| --- | --- | --- | --- |
| 1 | 'Parkinson disease' OR ' idiopathic parkinsonism' OR 'Lewy bodies of Parkinson disease' OR 'Lewy bodies of Parkinson`s disease' OR 'Lewy bodies of Parkinsons disease' OR 'Lewy body Parkinson disease' OR 'Lewy body Parkinson`s disease' OR 'Lewy body Parkinsons disease' OR 'paralysis agitans' OR 'Parkinson dementia complex' OR 'Parkinson`s disease' OR 'Parkinsons disease' OR 'primary parkinsonism' OR 'Parkinson disease' | 'idiopathic parkinsonism'/exp OR 'idiopathic parkinsonism' OR 'lewy bodies of parkinson disease'/exp OR 'lewy bodies of parkinson disease' OR 'lewy bodies of parkinson`s disease'/exp OR 'lewy bodies of parkinson`s disease' OR 'lewy bodies of parkinsons disease'/exp OR 'lewy bodies of parkinsons disease' OR 'lewy body parkinson disease'/exp OR 'lewy body parkinson disease' OR 'lewy body parkinson`s disease'/exp OR 'lewy body parkinson`s disease' OR 'lewy body parkinsons disease'/exp OR 'lewy body parkinsons disease' OR 'paralysis agitans'/exp OR 'paralysis agitans' OR 'parkinson dementia complex'/exp OR 'parkinson dementia complex' OR 'parkinson`s disease'/exp OR 'parkinson`s disease' OR 'parkinsons disease'/exp OR 'parkinsons disease' OR 'primary parkinsonism'/exp OR 'primary parkinsonism' OR 'parkinson disease'/exp OR 'parkinson disease' | 283572 |
| 2 | 'bipolar disorder' OR 'bipolar affective disorder' OR 'bipolar and related disorders' OR 'bipolar illness' OR 'bipolar psychosis' OR 'depression, manic' OR 'manic depression' OR 'manic depression psychosis' OR 'manic depressive' OR 'manic depressive disease' OR 'manic depressive disorder' OR 'manic depressive illness' OR 'manic depressive psychosis' OR 'manic depressive reaction' OR 'manic depressive syndrome' OR 'maniodepressive psychosis' OR 'mano depressive syndrome' OR 'psychosis, manic depressive' OR 'bipolar disorder' | 'bipolar affective disorder'/exp OR 'bipolar affective disorder' OR 'bipolar and related disorders'/exp OR 'bipolar and related disorders' OR 'bipolar illness'/exp OR 'bipolar illness' OR 'bipolar psychosis'/exp OR 'bipolar psychosis' OR 'depression, manic'/exp OR 'depression, manic' OR 'manic depression'/exp OR 'manic depression' OR 'manic depression psychosis'/exp OR 'manic depression psychosis' OR 'manic depressive'/exp OR 'manic depressive' OR 'manic depressive disease'/exp OR 'manic depressive disease' OR 'manic depressive disorder'/exp OR 'manic depressive disorder' OR 'manic depressive illness'/exp OR 'manic depressive illness' OR 'manic depressive psychosis'/exp OR 'manic depressive psychosis' OR 'manic depressive reaction'/exp OR 'manic depressive reaction' OR 'manic depressive syndrome'/exp OR 'manic depressive syndrome' OR 'maniodepressive psychosis'/exp OR 'maniodepressive psychosis' OR 'mano depressive syndrome'/exp OR 'mano depressive syndrome' OR 'psychosis, manic depressive'/exp OR 'psychosis, manic depressive' OR 'bipolar disorder'/exp OR 'bipolar disorder' | 112724 |
| 3 | #1 AND #2 | #1 AND #2 | 2260 |

**Cochrane**

| Search number | Query | Search Details | Results |
| --- | --- | --- | --- |
| 1 | (Parkinson disease) OR (Parkinson's Disease, Lewy Body) OR (Parkinsonism, Primary) OR (Parkinson's Disease Idiopathic Parkinson Disease) OR (Idiopathic Parkinson Disease) | [Parkinson Disease] explode all trees (Parkinson disease) OR (Parkinson's Disease, Lewy Body) OR (Parkinsonism, Primary) OR (Parkinson's Disease Idiopathic Parkinson Disease) OR (Idiopathic Parkinson Disease):ti,ab,kw | 16387 |
| 2 | ("bipolar disorder") OR (Type 2 Bipolar Disorder) OR (Bipolar Disorder Type 2) OR (Manic Disorder) OR (Disorder, Manic) | [Bipolar Disorder] explode all trees ("bipolar disorder") OR (Type 2 Bipolar Disorder) OR (Bipolar Disorder Type 2) OR (Manic Disorder) OR (Disorder, Manic) :ti,ab,kw | 9225 |
| 3 | #1 AND #2 | #1 AND #2 | 135 |
